# Functional organization of the human corpus callosum unveiled with BOLD-fMRI gradients

**DOI:** 10.1162/imag_a_00115

**Published:** 2024-03-25

**Authors:** Huan Huang, Yuchao Jiang, Hechun Li, Hanxi Wu, Xiaorong Feng, Jinnan Gong, Sisi Jiang, Dezhong Yao, Cheng Luo

**Affiliations:** The Clinical Hospital of Chengdu Brain Science Institute, MOE Key Lab for Neuroinformation, School of Life Science and Technology, University of Electronic Science and Technology of China, Chengdu, P. R. China; Research Unit of NeuroInformation, Chinese Academy of Medical Sciences, Chengdu, P. R. China; Institute of Science and Technology for Brain-Inspired Intelligence, Fudan University, Shanghai, China; Key Laboratory of Computational Neuroscience and Brain-Inspired Intelligence (Fudan University), Ministry of Education, Shanghai, China

**Keywords:** corpus callosum, functional connectivity, white matter BOLD fMRI, gradient mapping

## Abstract

Gradients capture the underlying functional organization of the brain. Cortical gradients have been well characterized, however very little is known about the underlying gradient of the white matter. Here, we proposed a functionally gradient mapping of the corpus callosum by using blood-oxygen-level-dependent functional magnetic resonance imaging (BOLD-fMRI), which for the first time uncovered three distinct but stable spatial axes: posterior-anterior, dorsal-ventral, and left-right. The three spatial patterns were replicated in another independent cohort and robust across scanning conditions. We further associated the three gradient maps with brain anatomy, connectome, and task-related brain functions, by using structural magnetic resonance imaging, both resting-state and task fMRI, and diffusion tensor imaging data. The posterior-anterior gradient distribution of the corpus callosum showed a similar pattern with the cerebral cortex, gradually extending from the primary cortex to the transmodal cortex. The dorsal-ventral gradient distribution revealed an N-shaped pattern from the primary cortex to the higher-order cognitive cortex. The posterior-anterior and dorsal-ventral gradient maps were also associated with white-matter microstructures, such as fractional anisotropy and myelin water fraction. The left-right gradient showed an inverted V-shaped pattern, which delineated the inter-hemisphere separation. These findings provide fundamental insight into the functional organization of the human corpus callosum, unveiling potential patterns of functional interaction with the cerebral cortex and their associations with cognitive behaviors.

## 1 Introduction

Neural information processing takes place in the superficial layer of the brain, within the gray matter. The synapses that connect different neurons in the gray matter are thought to be the main components of information processing. Moreover, the white matter—consisting primarily of myelinated axons—is conceptualized as the structural basis of human behaviors, and is increasingly recognized as being as critical for cognition as gray matter (Alexander-Bloch et al., 2013). The corpus callosum, with its vast assemblage of 200–300 million axons, is the largest white matter connectome in the human brain. It serves as a critical conduit that interconnects homologous regions in each of the two cerebral hemispheres (Tomasch, 1954). On the basis of its structural characteristics, the corpus callosum is likely fundamental for facilitating the interhemispheric communication of sensory, motor, and cognitive processes (Gazzaniga, 2000;Roland et al., 2017). The corpus callosum is also essential for the collaborative integration of functions that require the involvement of both cerebral hemispheres. Nevertheless, the majority of investigations have primarily focused on the structural aspects of the corpus callosum, with limited attention paid to its functional organization.

Evidence from diffusion tensor imaging (DTI) indicates that the corpus callosum has extensive connectivity with almost the entire cerebral cortex. Using DTI tractography, the corpus callosum can be divided into distinct subdivisions based on its trajectory to different cortical regions and functional networks (Huang et al., 2005;Y. Liu et al., 2021). Similarly, the organization of the corpus callosum can be divided by its functional connectivity into ten functional subregions, corresponding to different white matter functional networks (Wang et al., 2020). These findings support the idea that different corpus callosum subregions may be involved in distinct functions of the human brain. Recently, Friedrich et al. mapped a cortical principal gradient onto the corpus callosum midsection to explore the functional organization of the corpus callosum using a tractography approach (Friedrich et al., 2020). However, tractography analysis can produce inaccurate results (Jones & Cercignani, 2010), and the functional organization of the entire corpus callosum has not yet been fully explored.

The advent of functional magnetic resonance imaging (fMRI) has yielded unprecedented insights into the functional organization of the human brain in vivo. By detecting variations in blood-oxygen-level-dependent (BOLD) signals, numerous studies have identified functional circuits and distributed neural networks in the gray matter (Buckner & DiNicola, 2019;He et al., 2021;Huang et al., 2022;Power et al., 2011). In recent years, accumulating evidence suggests that changes in BOLD signals within the white matter may represent neural activity (G. J. Ji et al., 2017;G. J. Ji et al., 2019;Jiang et al., 2022), and that fMRI can reliably capture BOLD changes associated with external stimuli when appropriate analytical approaches are used. For example, fMRI activation has been detected within the corpus callosum during the performance of interhemispheric transfer tasks (Gawryluk et al., 2011), and neural-driven fluctuations have been reported in white matter signals during the resting state (Jiang et al., 2019). Notably, Jiang et al. documented white matter function–structure dissociation in patients with schizophrenia, indicating that the proper coupling of white matter function and structure is necessary to maintain healthy brain function (Jiang et al., 2021). These studies collectively suggest that investigations of white matter BOLD signals may further our understanding of the neural mechanisms underlying brain function.

Accumulating fMRI-derived evidence indicates that brain functional networks exhibit distinct time courses and correspond spatially to task-related functional activation (Power et al., 2011). This observation leads to the hypothesis that the functional organization of the brain may adhere to an inherent functional system. For example, Margulies et al. observed that cortical gradients formed by functional connectivity span from the sensorimotor network to the default mode network (DMN) (Margulies et al., 2016). These findings strongly align with existing models of cortical hierarchy in primates (Murray et al., 2014) and highlight the discrete spatial arrangements of brain regions that are implicated in various cognitive processes (Andersen & Cui, 2009;Mesulam, 1998). Additionally, these gradients may characterize the inherent organization patterns of the human cerebral cortex (Huntenburg et al., 2018). More recently, the cortical gradient method has also been used to study the functional organization of specific brain regions, including the thalamus (Yang et al., 2020), hippocampus (Vos de Wael et al., 2018), and insula (Royer et al., 2020). This emerging field will be useful for addressing the fundamental principles of cortical organization, studying structure–function cognition links, and examining cortical development and evolution (Bernhardt et al., 2022). Moreover, given that the function of the corpus callosum is defined by its connected cortex, it may be that the corpus callosum exhibits a similar functional organization.

The present study therefore aimed to provide a comprehensive map of the functional organization of the corpus callosum. First, we evaluated the functional connectivity between the corpus callosum and the cortex and applied the manifold learning method to generate gradient maps of the corpus callosum. Second, we analyzed the functional connectivity patterns between each callosal gradient and the cortex, aiming to elucidate the functional organization revealed by these distinct gradients. Third, we investigated the callosal functional gradient and its functional connectivity patterns with the cortex under task load conditions, and evaluated their associations with cognitive behavioral scores. Finally, we explored the relationship between callosal gradient and structure.

## 2 Materials and Methods

### 2.1 Data

Human neuroimaging data acquired as part of the Washington University-Minnesota Consortium Human Connectome Project (HCP) young adult publicly available dataset (Van Essen et al., 2013) were analyzed here. Participants were recruited from Washington University in St. Louis (St. Louis, MO) and the surrounding area. All participants gave informed consent. We selected 204 low-motion subjects (with no family relations) from the “1200 Subjects” HCP release (100 females, 104 males, age range, 22–36 years). These 204 participants were selected by excluding those with any fMRI run in which >50% of TRs had >0.25 mm framewise displacement (FD) or if a family relation was already included.

The datasets used in this study include fMRI, T1, and DTI data. (1) Two sessions (REST1 and REST2) of rest fMRI were acquired using multiband echo planar imaging (EPI) on a Siemens 3 T MR scanner (SKyra system). Each session comprised two runs (left-to-right and right-to-left phase encoding) of 14 min and 33 s each (repetition time (TR) = 720 ms, echo time (TE) = 33.1 ms, voxel dimension: 2-mm isotropic). The two runs were temporally concatenated for each session. The concatenation of the two different phase-encoded data ensured that any potential effect of phase encoding on gradient direction was counterbalanced by the opposing phase encoding. (2) Task fMRI data were acquired using the identical multiband EPI sequence as the rest fMRI session, and each session comprised two runs (left-to-right and right-to-left phase encoding). Similarly, the two runs were temporally concatenated for each session. However, the run duration ranged between 2 and 5 min depending on the specific task (7 tasks; EMOTION, GAMBLING, LANGUAGE, MOTOR, RELATIONAL, SOCIAL, and WORKING MEMORY). Data were collected over 2 d. On each day, 28 min of rest (eyes open with fixation) fMRI data across two runs were collected (56 min total), followed by 30 min of task fMRI data collection (60 min total). Details about the acquisition of rest fMRI (Smith et al., 2013) and task fMRI (Barch et al., 2013) were described elsewhere. The seven tasks were chosen to tap a broad range of cognitive and affective processes and activate a wide range of neural systems (Barch et al., 2013). (3) Structural T1-weighted data were acquired using a three-dimensional fast spoiled gradient echo sequence (TR = 6.008 ms, TE = 1.984 ms, matrix size = 256 × 256, field of view (FOV) = 256 × 256 mm^2^, slice thickness = 1 mm, no gap, and 152 slices). (4) The imaging parameters collecting diffusion data included a multiband factor of 3, nominal voxel size of 1.25 mm isotropic, 270 diffusion-weighted scans distributed equally over 3 shells (b values = 1,000, 2,000, and 3,000 s/mm^2^), and 18 volumes without diffusion weighting (b = 0 s/mm^2^). Participants who had complete data for each of the nine functional scans and diffusion scans were included. As a consequence, 17 of the original 204 individuals were excluded from the dataset, leaving 187 subjects for subsequent analysis.

To validate the findings from the HCP dataset, we included one independent normal cohort dataset: University of Electronic Science and Technology of China (UESTC) dataset. One hundred and twenty-two healthy controls (81 women, 38.0 ± 14.7 years old) were included in this study. High-resolution T1-weighted images and rest fMRI were collected on a 3.0 Tesla scanner (GE Discovery MR 750) with an 8-channel standard whole-head coil. During the scanning, participants were requested to keep their minds wandering during the resting-state scan with eyes open with fixation. Three-dimensional T1-weighted data were obtained by using a fast-spoiled gradient echo sequence (TR = 6.008 ms; TE = 1.984 ms; FOV = 256 × 256 mm^2^; matrix size = 256 × 256; slice thickness = 1 mm, no gap, and slice number = 156). Rest fMRI data were obtained with a gradient-echo echo-planar imaging (EPI) sequence (TR = 2,000 ms; TE = 30 ms; FOV = 24 × 24 cm^2^; matrix size = 64 × 64; slice thickness = 4 mm, no gap and slice number = 35). Scanning time lasted 510 s (255 volumes). All participants should be aware of the purpose as well as the procedure of our study and were required to provide written consent to be included in this study. The Ethics Committee of the Clinical Hospital of Chengdu Brain Science Institute approved the study protocol.

### 2.2 Data preprocessing

We used two software toolkits to preprocess the fMRI data, including the Data Processing Assistant for Resting-State fMRI (DPARSF) and SPM12. T1 images were firstly segmented into white-matter, gray-matter, and cerebrospinal fluid (CSF) and then normalized to the Montreal Neurological Institute (MNI) template. For the HCP dataset, considering that each fMRI session has two runs, the data of each run were preprocessed separately before being temporally concatenated. The initial preprocessing steps included: (1) Discarding the first 10 volumes for each run. (2) Slice-time correction. (3) Correcting for head motion-related signal changes. (4) Removal of linear trends to correct for signal drift. (5) Nuisance signal (including 24-parameter motion correction and the mean CSF signals) was regressed out. The 24 motion parameters included six rigid-body motion parameters (x, y, and z translations and rotations) and their values at the previous time point and the 12 corresponding squared values. (6) To avoid mixing white-matter and gray-matter signals, spatial smoothing was performed separately on the white-matter or gray-matter masks (G.-J. Ji et al., 2023). The individual functional images were smoothed (FWHM = 4 mm) separately on the two masks. (7) Spatially aligning to the MNI standard space (2 mm^3^) using the FNIRT nonlinear registration algorithm. For the UESTC dataset, same initial preprocessing steps were performed, including removing the first five volumes, slice timing correction, and realignment. Subjects with maximum motion >2 mm or 2° were excluded. The linear trend and nuisance signals were regressed. Then, the functional images were normalized onto the MNI space and resampled into 3 mm^3^.

The subsequent preprocessing steps of the two datasets are consistent. To retain as much of the signal of interest as possible, the temporal filtering was not performed. The white matter signal noise ratio (SNR) and sensitivity of the BOLD signal were lower compared with the cortex. Therefore, to enhance white matter SNR and suppress the effects of unstructured and autocorrelated noise that share similar temporal and spatial properties, the fMRI time series were reconstructed from a principal component analysis (PCA), whereby the components were filtered according to an empirically fitted Wishart organization to dampen the effects of noise (Glasser et al., 2016,^2018^). The Wishart filter was performed over all voxels for each individual separately in all datasets. In addition, for the task fMRI data, task block regressors were created for each task condition based on the time of onset, the duration, and the relative magnitude of each stimulus, and then convolved with hemodynamic response functions (HRF). The relevant regressors corresponding to each task were then regressed from the task fMRI data to ensure that functional connectivity was disambiguated from the potential confound of task co-activation effects (Cole et al., 2013). The residuals remaining from this regression were used for all subsequent analyses. For the HCP dataset, the fMRI time series were normalized to zero mean and unit standard deviation using Z-score. Finally, for each participant, we concatenated the data from their two runs into a session.

The FMRIB software library (FSL) (Jenkinson et al., 2012) was used to preprocess the diffusion data. Firstly, distortion correction (eddy currents, susceptibility-induced distortions, and subject’s motion) was conducted using eddy (Andersson & Sotiropoulos, 2016) with a field map estimated by*topup*. Further, diffusion tensors were fitted on the eddy-corrected data using*dtifit*. To facilitate comparison with the fMRI study, preprocessed diffusion data were normalized from the individual space to the standard space (MNI space) and resampled to 2 mm.

### 2.3 Mask delineation

A binary corpus callosum mask was delineated using two steps. Firstly, we used the JHU ICBM-DTI-81 WM atlas (Mori et al., 2008) to delineate binary masks for specific regions of the corpus callosum, including the genu, body, and splenium. Secondly, the masks for each subregion of the corpus callosum were merged to generate a binary mask for the entire corpus callosum. The final corpus callosum mask comprised a total of N = 4,401 voxels (35.2 cm^3^). A binary gray matter mask was delineated using the MNI152 probabilistic gray matter atlas. Voxels belonging to cortical gray matter and subcortex regions were included in the gray matter mask. fMRI data were consistently absent for a majority of individuals in a small proportion of voxels constituting the gray matter mask. After eliminating these voxels, the final gray matter mask comprised M = 164,360 voxels (1,314.9 cm^3^). Simultaneously, in the analysis of the UESTC dataset, the corpus callosum and gray matter masks were resampled to 3 mm^3^.

### 2.4 Functional connectivity and eigenmaps

Whole-brain functional connectivity was mapped for each voxel of the corpus callosum. Spatial gradients in the resulting maps were then computed to yield a continuous representation of functional connectivity variation across the corpus callosum (Fig. S1). Connectopic mapping was used to map spatial gradients for each individual, which involved computing a sequence of eigen decompositions to yield a Laplacian eigenmap (Belkin & Niyogi, 2003;Haak et al., 2018). Specifically, the temporally concatenated fMRI signals were represented for each individual in a matrix of dimension*T × N_g_*, where*T*denotes the number of time frames and*N_g_*denotes the number of gray matter voxels. Principal component analysis (PCA) was used to reduce the dimensionality of this matrix to*T × (T – 1)*(Tian et al., 2020). The fMRI signal at each corpus callosum voxel was then correlated (Pearson correlation) with each column of the PCA-transformed matrix, resulting in a connectivity matrix of dimension*N_c_× (T – 1)*, where*N_c_*denotes the number of callosal voxels. Correlation coefficients were r-to-z transformed using the Fisher transformation. Each row of this matrix provided a connectional fingerprint for a particular corpus callosum voxel in the PCA-transformed space. The similarity in the connectional fingerprints between each pair of callosal voxels was then quantified with the*η^2^*coefficient, which resulted in a symmetric matrix of dimension*N_c_× N_c_*for each individual. Then, similarity matrices were averaged across all individuals, and the group-averaged similarity matrix was transformed into a sparse graph using the weighted adjacency matrix, whereby the weights of all connectivity with a Euclidean distance less than*ε*were set to zero. The connection density was determined by the smallest value of*ε*that ensured a connected graph (Fornito et al., 2013). The Laplacian matrix,*L*, was then computed according to*L=D – W*, where*D*denotes the diagonal matrix of node strengths and*W*denotes the sparse adjacency matrix. Finally, the eigenvectors and eigenvalues of the Laplacian matrix were computed. The smallest eigenvalue was necessarily zero, while all other eigenvalues were positive due to the connectedness of the graph (Fig. S2). The eigenvector with a zero eigenvalue was a constant and discarded. The eigenvectors with the second, third, and fourth smallest eigenvalue were called principal, secondary, and third gradients respectively, and each eigenvector accounts for the highest variability possible under the constraint that all eigenvectors are orthogonal to each other. These three largest eigenvectors (Fig. S1) explained a lot more variance in the Laplacian matrix than the rest eigenvectors, which were thus given no further consideration here. In this study, each gradient characterized a continuous mode of spatial variation in functional connectivity across the spatial extent of the corpus callosum. The*N_c_*values defining each gradient were projected onto the three-dimensional (3D) anatomy of the corpus callosum in MNI standard space for visualization and further analyses (Fig. S1). The gradient analysis was performed in all fMRI scanning conditions.

### 2.5 Spatial distribution of the callosal functional connectivity gradient

To explore how the functional gradient varies along the spatial positions of the corpus callosum, we generated gradient functions along the main axes of a callosal structure using an automatic procedure that moved from the image axes to the region of interest (ROI)-based axes, and then sampled the gradient values as a function of their position along them (Drori et al., 2022). The algorithm first calculated the singular value decomposition of the callosal voxel three-dimensional image coordinates to find the three main orthogonal axes of the structure. Second, for each of the three main axes, we segmented the ROIs with equal spacing. For this purpose, we defined the data edge as the two hyperplanes delineated by the two extreme data points of the data for the axis and defined the axis as the normal to the plane. Next, the data were segmented by n−1 parallel hyperplanes, equally spaced between the two data edges. Voxels were then classified into n segments based on the criteria of their distance from the planes. In our study, we chose to use n = 5. The flow diagram was shown in the supplementary materials (Fig. S3).

On the basis of previous findings (Reyes et al., 2020), we inferred that the spatial distribution of the principal gradient corresponded strongly to the underlying structural properties. Using two corpus callosum atlases, a JHU ICBM-DTI-81 white matter atlas based on diffusion MRI (Mori et al., 2008) and a Witelson atlas (Witelson, 1989), we applied k-means clustering (where k = 3 and 7, based on the JHU and Witelson atlases, respectively) to the principal gradient, and assessed their relationship with the corpus callosum atlas by computing a mean Dice coefficient between the clusters and subparts of the atlas. To further evaluate the significance of the mean Dice coefficient, a permutation test was performed. Specifically, cluster labels were randomly permuted 1,000 times to generate the null organization. The p-value was then calculated based on the position of the actual mean Dice coefficient within the null organization. The significance level was set at p < 0.05. To investigate whether gradients were related to the intrinsic spatial distance of the corpus callosum, we calculated the Euclidean distance between the peak voxel of the gradient map and the remaining voxels. We then corrected the estimated p-value in the voxel-wise Pearson’s correlations between the gradients and spatial (Euclidean) distances.

### 2.6 Functional connectivity between the corpus callosum and cortex along the gradient

Because the gradient reveals the continuous functional pattern of the corpus callosum, functional connectivity along all three gradients was calculated. For each gradient, the gradient map was binned into 20-percentile increments and binarized, resulting in five ROIs ranging from 0%–20% to 80%–100%. The corresponding cortical function pattern was then established for each callosal ROI. The cortex was segmented into 400 ROIs using the Schaefer 400 atlas, as reported in a previous study (Schaefer et al., 2018). For each subject, the representative time series of each ROI was obtained by averaging the fMRI time series across all voxels in the ROI. Next, we calculated the Pearson’s correlation between the corpus callosum and cortex. A 5*×*400 functional connection matrix between the corpus callosum and cortex was obtained for each callosal gradient. This matrix both quantified the intensity of the association between gradient-specific corpus callosum subregions and the cortex, and characterized the corresponding functional attributes of different callosal subregions. All calculations with Pearson’s correlations used Fisher’s z-transformed values, which were reconverted to r-values for reporting purposes. Note that calculations were performed on data from all scanning conditions.

### 2.7 Correlations between callosal functional connectivity and cognitive traits

The corpus callosum acts as a hub structure for signal transmission between the left and right hemispheres. Its functional connectivity with the cortex may thus contain idiosyncratic information that may be linked to cognitive traits, especially under task-loaded conditions. To test for cross-condition differences in functional connectivity–behavior relationships, we calculated voxel-wide univariate correlations between callosal functional connectivity in different scanning conditions and behavioral scores.

#### 2.7.1 Cognitive data

The 10 cognitive subdomains tested in the HCP were considered: episodic memory, executive functions, fluid intelligence, language, processing speed, self-regulation/impulsivity, spatial orientation, sustained visual attention, verbal episodic memory, and working memory (Barch et al., 2013). For subdomains with multiple unadjusted original scores, a single score was obtained by projecting the data from the PCA onto the first component (seeTable S2for the full list) (Griffa et al., 2022). Each participant’s component score for each principal component was used as the behavioral measure of interest.

#### 2.7.2 Callosal functional connectivity–behavior correlations

We first calculated the functional connectivity of each callosal voxel to the cortex. To deal with potential intercorrelations of functional connectivity for each callosal voxel, we performed PCA on the functional connectivity between the callosal voxel and cortex to yield one latent variable per callosal voxel; this indicated the callosal voxel’s weighting within the cortical functional organization. Next, we calculated Pearson’s correlation coefficients between 44,010 (4401 voxels × 10 behavioral variables) pairs of individual callosal–cortical functional connectivity scores and behavioral scores across the subjects in different scanning conditions. Differences in callosum–behavior correlations between different scanning conditions were assessed using t-tests of absolute r-values across the various pairs of conditions. The absolute r-values accounted for both positive and negative correlations between callosal functional connectivity and behavioral scores.

### 2.8 Assessing the relationship between callosal gradients and structure

The corpus callosum is the largest commissural fiber bundle in humans; it primarily consists of myelinated axons with varying diameters. Myelin sheaths surround axons and are essential for the efficient conduction of action potentials between neurons. In an attempt to reveal the potential relationship between function and structure, we analyzed the correlations between the callosal functional gradient and fiber bundle structure and myelination. First, we examined fractional anisotropy (FA) to characterize the fiber bundle structural properties of the corpus callosum using the FMRIB software library. Pearson’s correlation analysis was used to examine voxel-wise associations between group-averaged FA values and three group-level callosal gradients. Second, we downloaded the recently released average myelin water fraction (MWF) organization map of the adult human brain to assess the correlations between myelin in the corpus callosum and the three functional gradients (H. Liu et al., 2019). All correlations between gradients and other markers were compared across the gradients using Steiger’s test. Because the sign of the gradient is arbitrary, we flipped the signs of gradients with negative correlations, resulting in only positive correlations. This provided more conservative significance testing results because the differences between correlation values were decreased or remained the same.

### 2.9 Reliability and reproducibility assessment

To estimate the reliability and reproducibility of the callosal gradients, we first evaluated the effect of the number of dimensionality reduction in grey matter time series on the gradient (Fig. S4). Subsequently, we evaluated the gradient consistency across scanning conditions in the main dataset and assessed reproducibility in two other retest datasets. To assess the gradient consistency in the resting scan conditions, we estimated the spatial correlations between the corresponding gradients in REST1 and REST2. To estimate the stability of gradients, the spatial correlations between the gradients corresponding to the resting and task scanning conditions in the main dataset were calculated. Reproducibility was determined by re-calculating results in the UESTC dataset and a random sample of half of the subjects from the main dataset (rHCP, n = 93 subjects). Specifically, we calculated the spatial correlations of gradients between the discovery and validation datasets.

## 3 Results

### 3.1 Functional gradients of the corpus callosum

Whole-brain functional connectivity was calculated for each corpus callosum voxel and individual. Laplacian eigenmaps were used to calculate the spatial gradients characterizing the continuous patterns of functional connectivity variation across the topography of the corpus callosum (Fig. S1). At the group level, the eigenvectors with the second-, third-, and fourth-smallest eigenvalues were named the principal, second, and third gradients, respectively; each eigenvector accounted for the highest variability possible, under the constraint that all eigenvectors were orthogonal to each other. The first three gradients were analyzed because they explained 27% of the variance and corresponded to the clearest elbow in the scree plot. Further components explained substantially less variance and were therefore not considered (Fig. S1). Note that, because the group-level gradients under different scanning conditions were extremely similar (r > 0.95) (Table S1), all described gradients are from the REST1 scanning condition unless otherwise indicated. A gradient map of the different scanning conditions is provided inFigure S5. The principal gradient accounted for 18% of the variance and captured the main axis of the macroscale functional organization of the corpus callosum. The additional orthogonal components (second and third gradients) accounted for 4%–5% of the variance, and each showed additional functional organizational properties. Specifically, the principal gradient extended from the posterior aspects of the corpus callosum splenium to the anterior corpus callosum genu, whereas the second and third gradients encompassed the dorsal–ventral (DV) and left–right (LR) extensions of the corpus callosum, respectively.

### 3.2 Spatial distribution of the callosal functional connectivity gradients

**Fig. 1.**
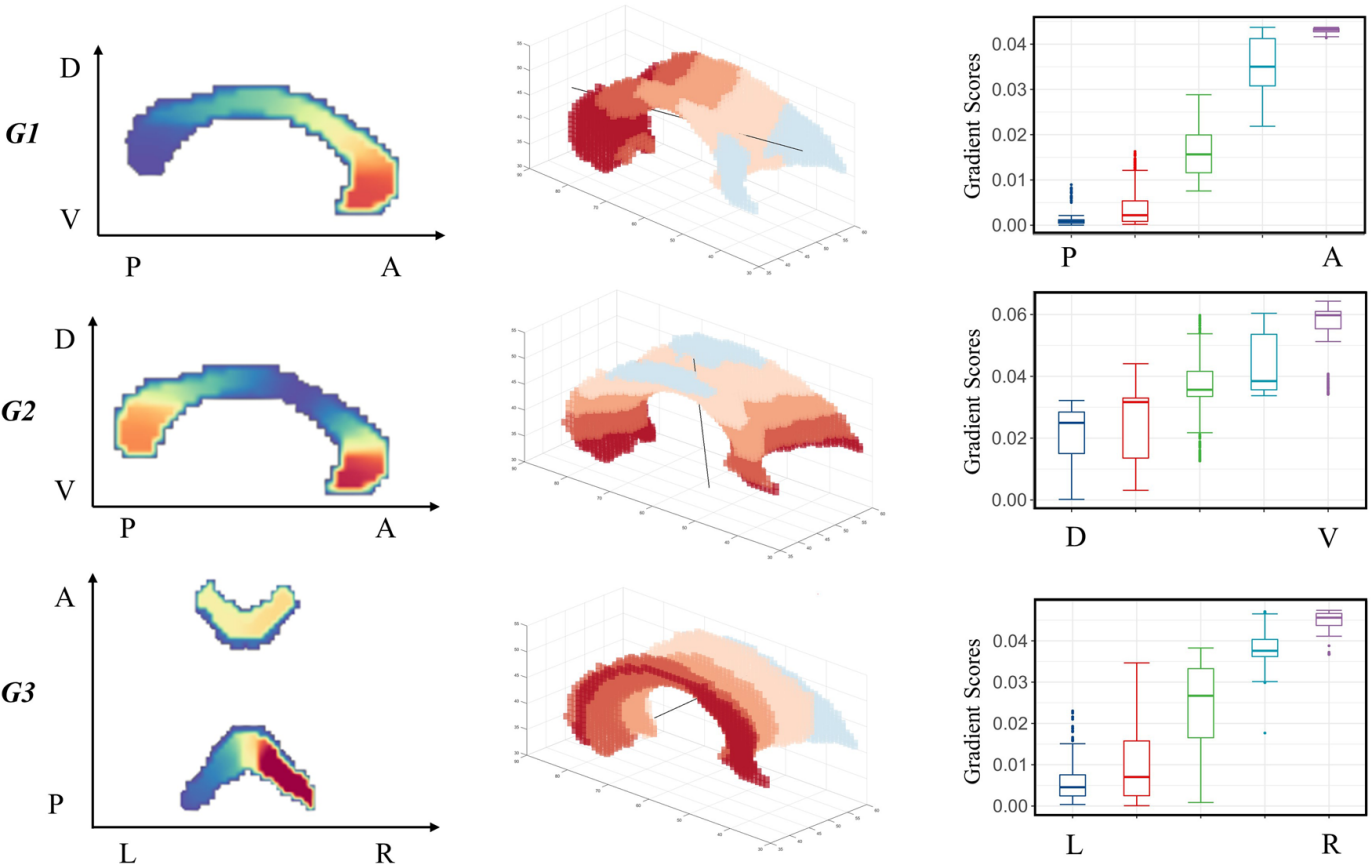
Automatic computation of the callosal three main axes in the callosal gradients. The G1 extended along the posterior-to-anterior (PA) axis, the G2 extended along the dorsal-to-ventral (DV), and the G3 extended along the left-to-right (LR) axis. The gradient values increase monotonically along the axis, and there are significant differences between the gradient values of adjacent ROIs (p < 0.05, FWE corrected). G1, principal gradient; G2, secondary gradient; G3, third gradient.

To assess spatial variability in the gradients, we used an automatic tool to express the main axes of the corpus callosum at the group level. To find the main orthogonal axes of the corpus callosum, we used a mask of the corpus callosum and performed singular value decomposition of the callosal voxel three-dimensional image coordinates to find the three main orthogonal axes on the voxel coordinates. The voxels were then divided into five equally spaced segments along each of the axes, and the mean value of the gradient was calculated for each segment, yielding a quantitative function along each axis.Figure 1shows that, in the corpus callosum, the three main axes roughly corresponded to ROIs of the posterior–anterior (PA), DV, and LR axes. These gradients carried significant change along the main axes of the callosal structures. Notably, the gradients exhibited spatial organization patterns corresponding to the three main axes. The principal gradient corresponded to the PA axis, the secondary gradient corresponded to the DV axis, and the third gradient corresponded to the LR axis of the corpus callosum; all three showed progressive patterns, with significant differences along each axis (p < 0.05).

**Fig. 2.**
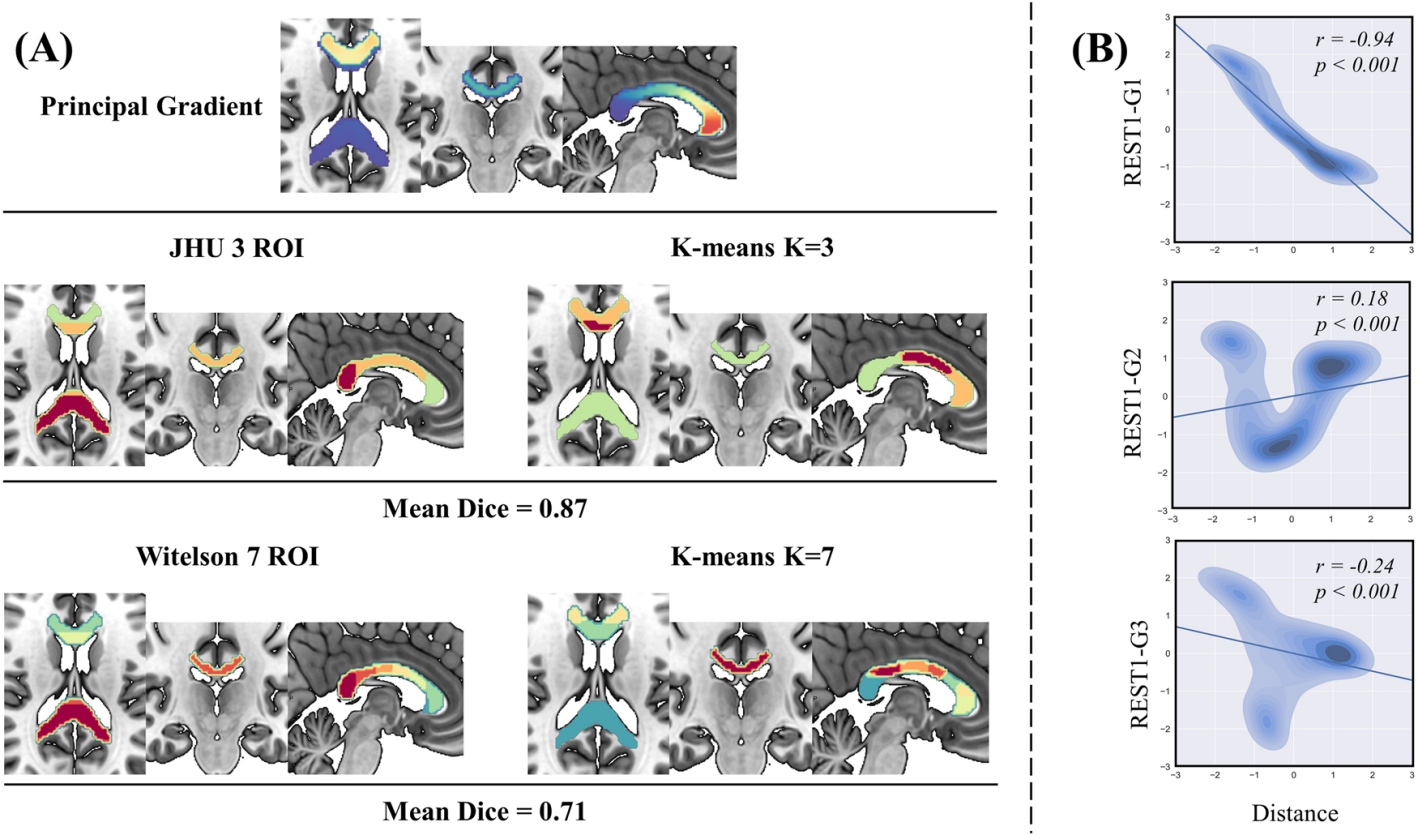
The spatial distribution of the callosal functional gradient. (A) The k-means clustering was applied to the principal gradient (first row), with the number of clusters set to three (second row) and seven (third row) based on the JHU and Witelson atlas respectively. Dice indices denote geometric overlaps between k-means clusters of callosal functional principal gradient and the callosal anatomical templates. (B) Associations between the callosal gradients and intrinsic geometry. The principal gradient (r = -0.94, p < 0.001), secondary gradient (r = 0.18, p < 0.001), and third gradient (r = -0.24, p < 0.001) show prominent correlations with thalamic Euclidean distance, which indicated the gradients were closely related to the intrinsic geometry of the corpus callosum.

Consistent with previous studies (Tian et al., 2020;Vos de Wael et al., 2018;Yang et al., 2020), the functional principal gradient closely corresponded to the underlying anatomy, both in a continuous sense and after applying k-means clustering (Fig. 2A); the resulting parcellation recapitulated the anatomical boundaries of the genu, body, and splenium of the corpus callosum (Fig. 2A; mean Dice coefficient = 0.87 overall subdivisions, permuted p < 0.05). Further, using the more delicate Witelson atlas, the mean Dice coefficient of k-means clustering was 0.71 (permuted p < 0.05), indicating that the principal gradient had a similar subdivision to the morphometry-based gross subdivision. In addition, we observed lower gradient scores with increased spatial distance in both the principal (r = −0.94, p < 0.001;Fig. 2B, top) and third (r = −0.24, p < 0.001;Fig. 2B, bottom) gradients. By contrast, in the secondary gradient, gradient scores increased with increased spatial distance (r = 0.18, p < 0.001;Fig. 2B, middle). These results indicate that the spatial distributions of the gradients are closely related to the intrinsic geometry of the corpus callosum.

### 3.3 Gradual functional connectivity patterns within the corpus callosum

We applied connectopic mapping to the resting-state and seven-task fMRI data to estimate the callosal–cortical functional connectivity patterns in the corpus callosum. At the group level, the gradients followed the PA, DV, or LR trajectories. To visualize the average change in connectivity, we conducted a group-level analysis in which we estimated the ROI-based connectivity (Pearson’s correlations) with the cortex in various subregions along the main axis. For each gradient, the gradient map was binned into 20-percentile decrements and binarized, resulting in five ROIs.

#### 3.3.1 Functional connectivity along the principal gradient

**Fig. 3.**
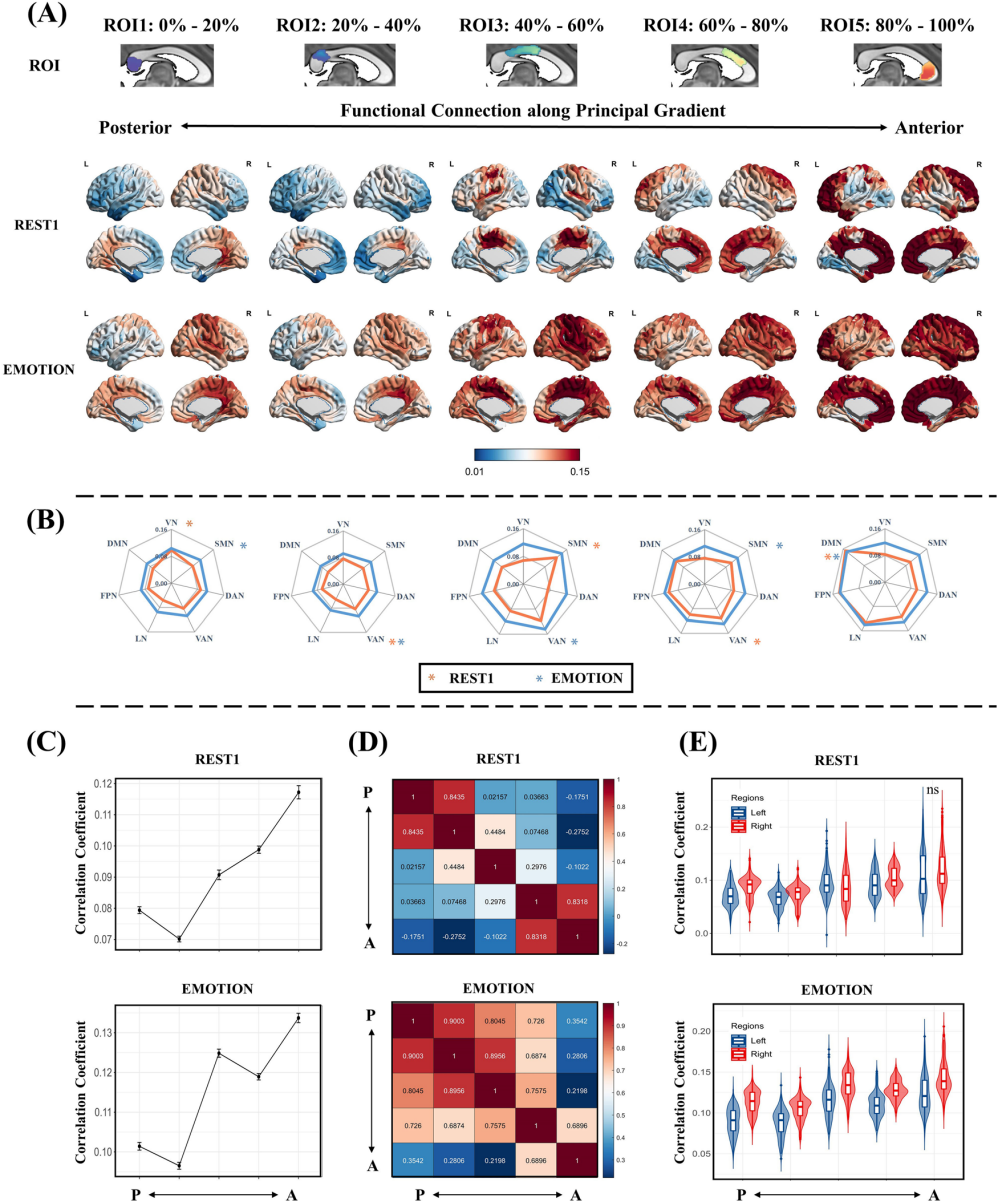
Callosal connectivity patterns along the callosal principal gradient. (A) Functional connectivity maps based on the ROIs (first row) along the callosal principal gradient in REST1 (second row) and EMOTION (third row) scanning conditions. These ROIs were defined according to the principal gradient scores, resulting in 5 ROIs ranging from 0%–20% to 80%–100%. The black arrows represent the approximate location of the ROIs along the callosal posterior-anterior (PA) axis. (B) Averaging the callosal-cortical functional connectivity maps within 7 intrinsic functional networks. The asterisk indicates the network with the strongest functional connectivity. The orange and blue colors indicate REST1 and EMOTION scanning conditions, respectively. (C) The average functional connectivity between the ROIs along the callosal functional principal gradient and the cortical. (D) The similarity between the functional connectivity maps is based on the ROIs along the callosal principal gradient (Pearson correlations). (E) The average functional connectivity between the ROIs along the callosal functional principal gradient and the left and right cortical. The ‘ns’ means no significant difference and significant differences exist between the other unlabeled groups (p < 0.05, FWE corrected). VN, visual network; SMN, somatomotor network; DAN, dorsal attention network; VAN, ventral attention network; LN, limbic network; FPN, frontoparietal network; DMN, default mode network.

Figure 3shows the projections of the five ROIs along the principal gradient under REST1 and EMOTION scanning conditions. The principal gradient exhibited a gradual pattern of connectivity changes with the cortex, transitioning from regions involved in visual processing (visual network) to those associated with sensory processing (somatomotor and ventral attention networks), and finally to areas linked to higher-order conceptual representations (DMN and frontoparietal and limbic networks) (Fig. 3B). Crucially, compared with that of the resting state, task-state functional connectivity between the corpus callosum and cortex was significantly increased along the PA axis. However, the overall pattern of connectivity remained consistent between the resting and task states, indicating that the functional division of posterior and anterior regions was present during both states (Fig. 3B). The results under other scanning conditions are included inFigure S6. The average functional connectivity between the corpus callosum and cortex was increased during task-state scanning conditions, suggesting that the interplay between the corpus callosum and the cortex might be strengthened under higher cognitive task demands (Fig. 6A). Furthermore, along the principal gradient, functional connectivity between the corpus callosum and cortex increased progressively from posterior to anterior regions (Fig. 3C); similar trends were observed across various scanning conditions (Fig. S9A).

To quantify the changes in functional connectivity patterns between the corpus callosum and cortex, we calculated Pearson’s correlation coefficients for the functional connectivity projection map of each ROI from the principal gradient. The projections of each ROI exhibited the greatest similarity with those of neighboring ROIs during both resting-state and EMOTION scanning conditions (Fig. 3D). These findings suggest that the functional connectivity pattern does not occur randomly, but that it follows a continuous process of gradual change. Furthermore, given the important role of the corpus callosum in facilitating the transmission of functional activities between left and right hemispheres, we evaluated the functional connectivity of the cortex in both hemispheres, with each ROI derived from the principal gradient. This analysis revealed that functional connectivity to the right hemisphere was notably stronger compared with that to the left hemisphere for each ROI; this was evident in both resting-state and EMOTION scanning conditions (Fig. 3E), and consistent results were also detected under other scanning conditions (Fig. S10A).

#### 3.3.2 Functional connectivity along the secondary gradient

**Fig. 4.**
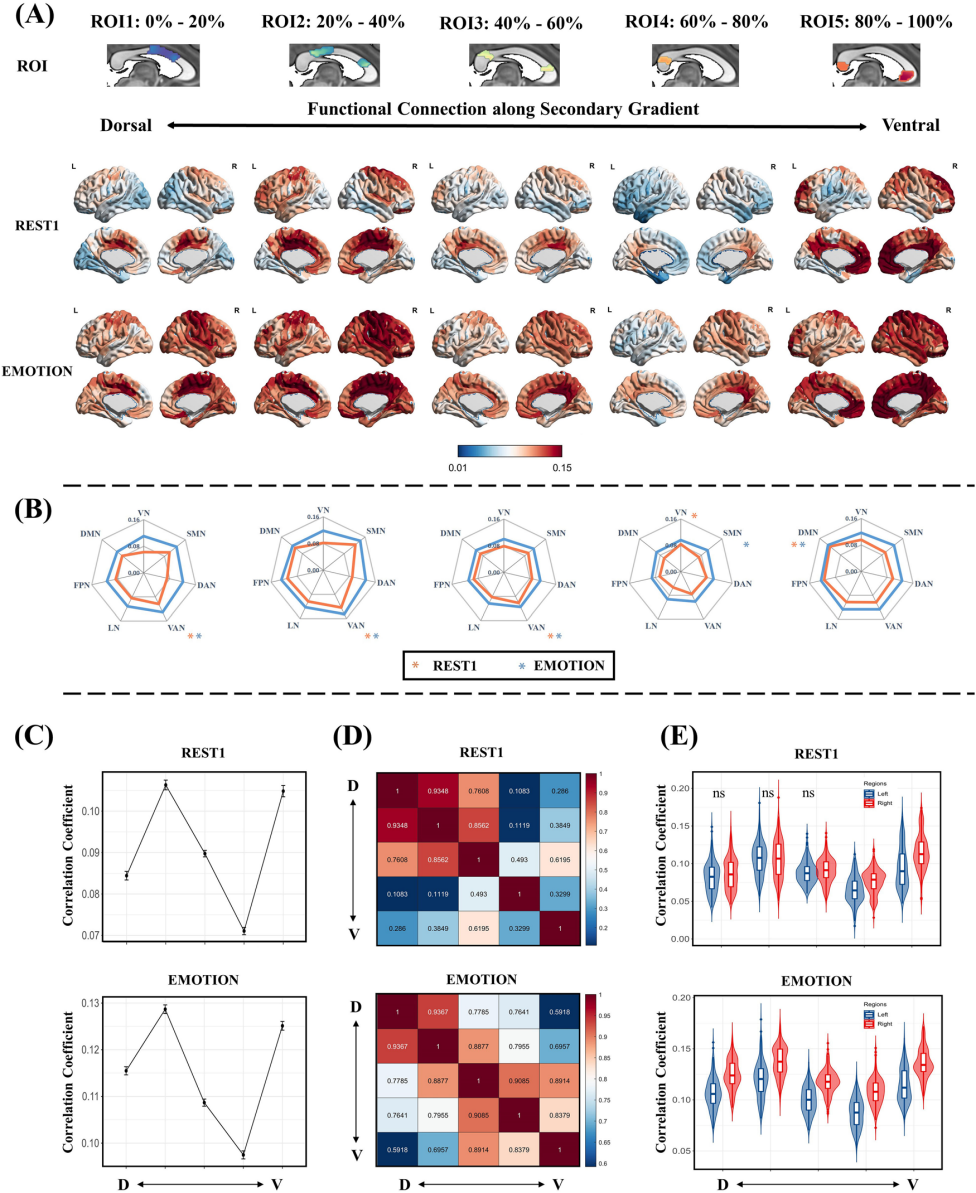
Callosal connectivity patterns along the callosal secondary gradient. (A) Functional connectivity maps based on the ROIs (first row) along the callosal secondary gradient in REST1 (second row) and EMOTION (third row) scanning conditions. These ROIs were defined according to the secondary gradient scores, resulting in 5 ROIs ranging from 0%–20% to 80%–100%. The black arrows represent the approximate location of the ROIs along the callosal dorsal-ventral (DV) axis. (B) Averaging the callosal-cortical functional connectivity maps within 7 intrinsic functional networks. The asterisk indicates the network with the strongest functional connectivity. The orange and blue colors indicate REST1 and EMOTION scanning conditions, respectively. (C) The average functional connectivity between the ROIs along the callosal functional secondary gradient and the cortical. (D) The similarity between the functional connectivity maps is based on the ROIs along the callosal secondary gradient (Pearson correlations). (E) The average functional connectivity between the ROIs along the callosal functional secondary gradient and the left and right cortical. The ‘ns’ means no significant difference and significant differences exist between the other unlabeled groups (p < 0.05, FWE corrected). VN, visual network; SMN, somatomotor network; DAN, dorsal attention network; VAN, ventral attention network; LN, limbic network; FPN, frontoparietal network; DMN, default mode network.

Figure 4shows the projections of the five ROIs along the secondary gradient under REST1 and EMOTION scanning conditions. Similar to the principal gradient, the secondary gradient exhibited a gradual shift in connectivity with the cortex, which transitioned from connectivity with regions linked to sensory processing (ventral attention and somatomotor networks) to those involved in vision (visual network), and finally to higher-order networks (DMN and frontoparietal and limbic networks) (Fig. 4B). Along the DV axis, functional connectivity between the corpus callosum and cortex was significantly increased in the task state compared with the resting state, although a consistent overall pattern was maintained. Results from other scanning conditions are shown inFigure S7. We also observed a significant increase in mean functional connectivity to the cortex for each ROI of the secondary gradient during task-state conditions (Fig. 6B). Along the secondary gradient, functional connectivity between the corpus callosum and cortex generally increased from ventral to dorsal regions followed by a decrease and a subsequent increase, thus forming an “N”-shaped pattern (Fig. 4C). Similar trends were detected under different scanning conditions (Fig. S9B).

The correlation results revealed that, under both resting-state and EMOTION scanning conditions (Fig. 4D), the projections of most ROIs in the secondary gradient exhibited the greatest similarity with neighboring ROI projections. These results indicate that functional connectivity patterns along the secondary gradient change in a specific pattern. Furthermore, along the secondary gradient, functional connectivity to the right cortex was significantly greater than that to the left cortex under both resting-state and EMOTION scanning conditions (Fig. 4E). Consistent results were also observed under different scanning conditions (Fig. S10B).

#### 3.3.3 Functional connectivity along the third gradient

**Fig. 5.**
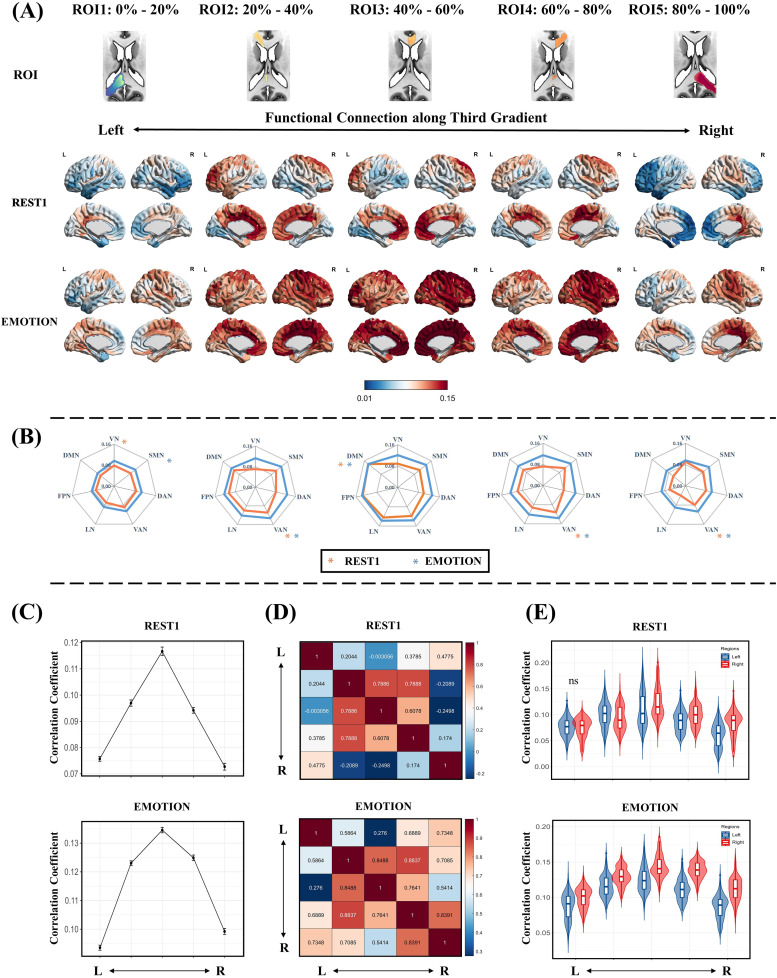
Callosal connectivity patterns along the callosal third gradient. (A) Functional connectivity maps based on the ROIs (first row) along the callosal third gradient in REST1 (second row) and EMOTION (third row) scanning conditions. These ROIs were defined according to the third gradient scores, resulting in 5 ROIs ranging from 0%–20% to 80%–100%. The black arrows represent the approximate location of the ROIs along the callosal left-right (RL) axis. (B) Averaging the callosal-cortical functional connectivity maps within 7 intrinsic functional networks. The asterisk indicates the network with the strongest functional connectivity. The Orange and blue colors indicate REST1 and EMOTION scanning conditions, respectively. (C) The average functional connectivity between the ROIs along the callosal functional third gradient and the cortical. (D) The similarity between the functional connectivity maps is based on the ROIs along the callosal third gradient (Pearson correlations). (E) The average functional connectivity between the ROIs along the callosal functional third gradient and the left and right cortical. The ‘ns’ means no significant difference and significant differences exist between the other unlabeled groups (p < 0.05, FWE corrected). VN, visual network; SMN, somatomotor network; DAN, dorsal attention network; VAN, ventral attention network; LN, limbic network; FPN, frontoparietal network; DMN, default mode network.

**Fig. 6.**
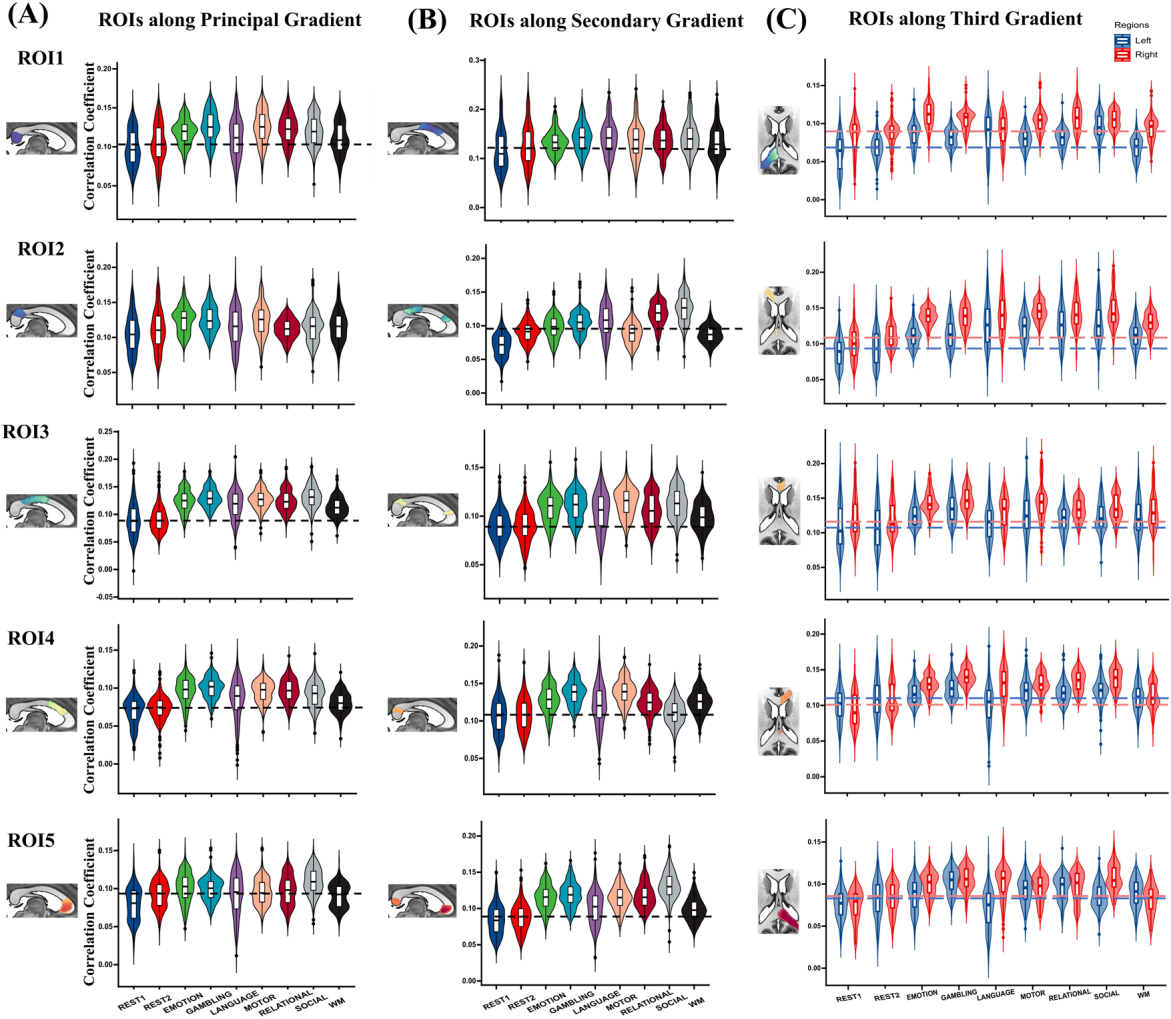
Functional connectivity maps based on the ROIs along the callosal principal gradient (A), secondary gradient (B), and third gradient (C) across different scanning conditions. Each row shows the functional connectivity across different scanning conditions for the five callosal ROIs, ranging from 0%–20% to 80%–100% gradient scores. The black dotted line indicates the maximum value of the mean value of functional connectivity in REST1 and REST2. The blue and red dashed lines represent the maximum values of the mean functional connectivity to the left and right cortex in REST1 and REST2, respectively. The results showed that in most cases, the functional connectivity of the corpus callosum in the task state was significantly greater than that in the resting state.

Figure 5shows the projections of the five ROIs along the third gradient under REST1 and EMOTION scanning conditions. The third gradient appeared to reflect connectivity changes in the LR direction between the corpus callosum and the cortex, transitioning from regions associated with visual processing (visual network), sensory processing (somatomotor network), and attention function (ventral attention networks) to higher-order networks (DMN and frontoparietal and limbic networks), and then back to areas related to attentional function (ventral attention network) (Fig. 5B). Similarly, along the LR axis, functional connectivity between the corpus callosum and cortex increased significantly under different scanning conditions, although the overall connectivity pattern remained consistent. The results from other scanning conditions are shown inFigure S8. For each ROI of the third gradient, the mean functional connectivity between the corpus callosum and the left and right hemispheres was significantly increased under task-state conditions (Fig. 6C). Along the third gradient, functional connectivity between the corpus callosum and cortex generally increased and then decreased from left to right, reaching a peak at the central sagittal plane of the corpus callosum (the third ROI of the third gradient). This pattern showed an inverted triangle shape with left and right symmetry (Fig. 5C); similar trends were detected under different scanning conditions (Fig. S9C).

Unlike for the principal and secondary gradients, correlation results revealed that, under both resting and EMOTION scanning conditions, the projections of each ROI in the third gradient were more similar to those of the symmetrical ROI in the opposite hemisphere (Fig. 5D). The functional connectivity pattern along the third gradient presented a similar LR pattern. Importantly, the similarity between all ROIs was significantly increased under task conditions compared to the resting state. Along the third gradient, under REST1 scanning conditions, functional connectivity between the right callosal ROIs (the first and second ROIs of the third gradient) and the right cortex was significantly greater than that of the left hemisphere. By contrast, functional connectivity between the left callosal ROIs (the fourth and fifth ROIs of the third gradient) and the left cortex was significantly greater than that of the right hemisphere. However, in the EMOTION scanning condition, functional connectivity between all ROIs and the right cortex was greater than that of the left cortex (Fig. 5E). Consistent results were detected under other scanning conditions (Fig. S10C). Our results thus suggest the existence of hemispheric laterality in functional connectivity between the corpus callosum and the cortex and indicate that task conditions may more effectively induce this hemispheric laterality.

### 3.4 Correlations between callosal functional connectivity and cognitive traits

**Fig. 7.**
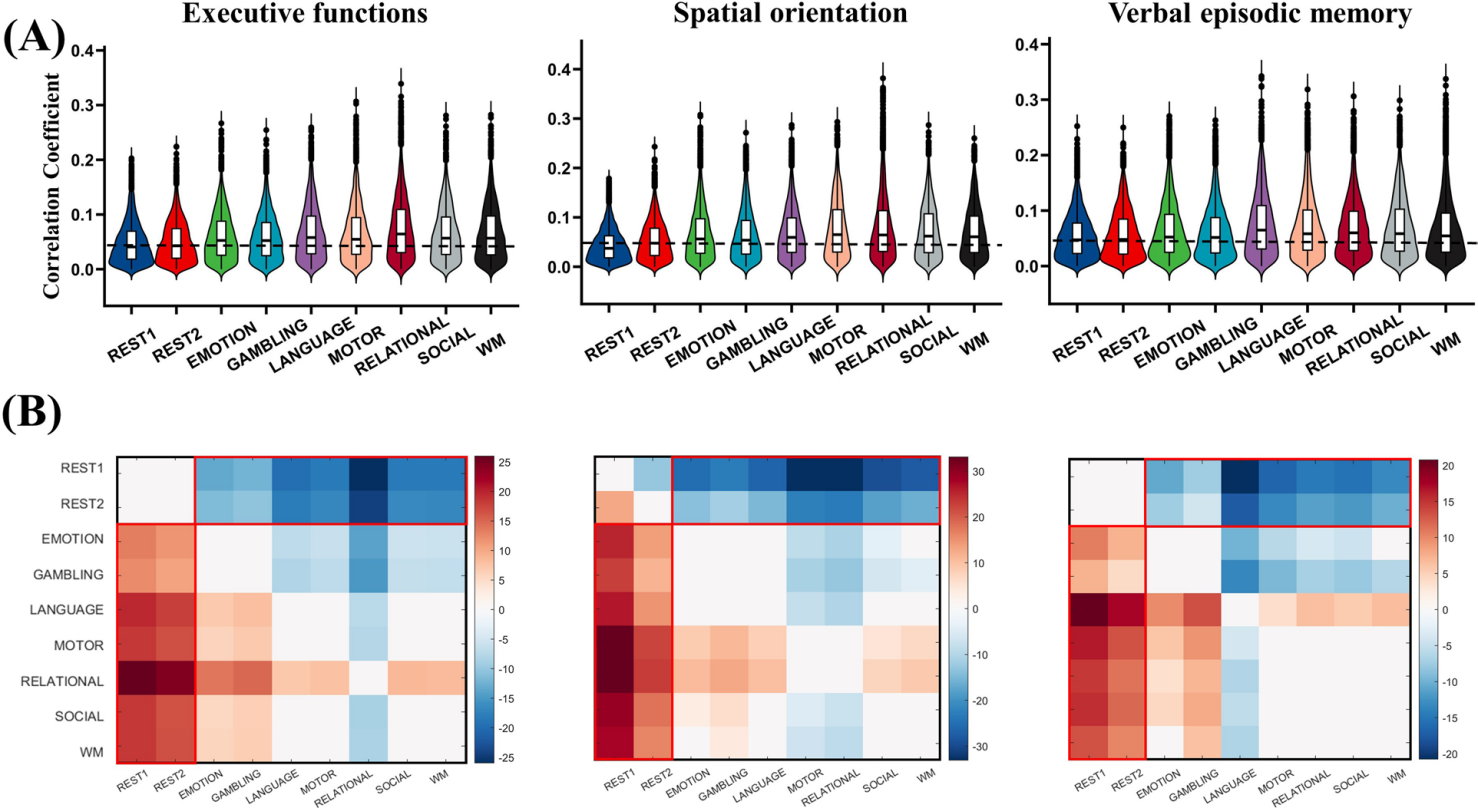
Relating callosal-cortical functional connectivity to behavioral scores in all scanning conditions. (A) Correlations between callosal functional connectivity and executive functions, spatial orientation, and verbal episodic memory composite (PCA-based) behavioral scores were shown. The correlation results for other behavioral scores are in the supplementary material. Absolute values of Pearson’s correlation coefficients are shown for each callosal voxel. The black dotted line indicates the maximum value of the averaged correlation in REST1 and REST2. (B) The T-value map was obtained by a paired-sample T-test for correlation across all scanning conditions. The results showed that the functional connectivity between the corpus callosum and the cortex was more strongly correlated with the behavior in the task scanning conditions.

We calculated the Pearson’s correlation coefficient between callosal functional connectivity with composite (PCA-based) cognitive scores at each callosal voxel. We focused on the overall correlation between the callosal-cortex functional connectivity and behavior, so we did not correct for multiple comparisons in this analysis. At the voxel level, connectivity–behavior correlations for both resting and task scanning conditions were low (maximum r = 0.39 for task, r = 0.30 for resting).Figure 7Ashows the correlations between functional connectivity of the corpus callosum and the cortex and executive function, spatial orientation, and verbal episodic memory scores. The results of other cognitive scores are shown inFigure S11. In almost all cases, correlations between absolute r-values were significantly stronger for task conditions than for resting conditions. More details are provided inFigure S12. Although there were no significant differences within the resting-state scanning conditions, there was a wide range of significant differences between the resting and task states (p < 0.05, family-wise error corrected). Moreover, there were significant differences among different task states (Fig. 7B), suggesting that the functional activities of the corpus callosum may differ across different task states.

### 3.5 Callosal gradients reflect callosal structural properties

**Fig. 8.**
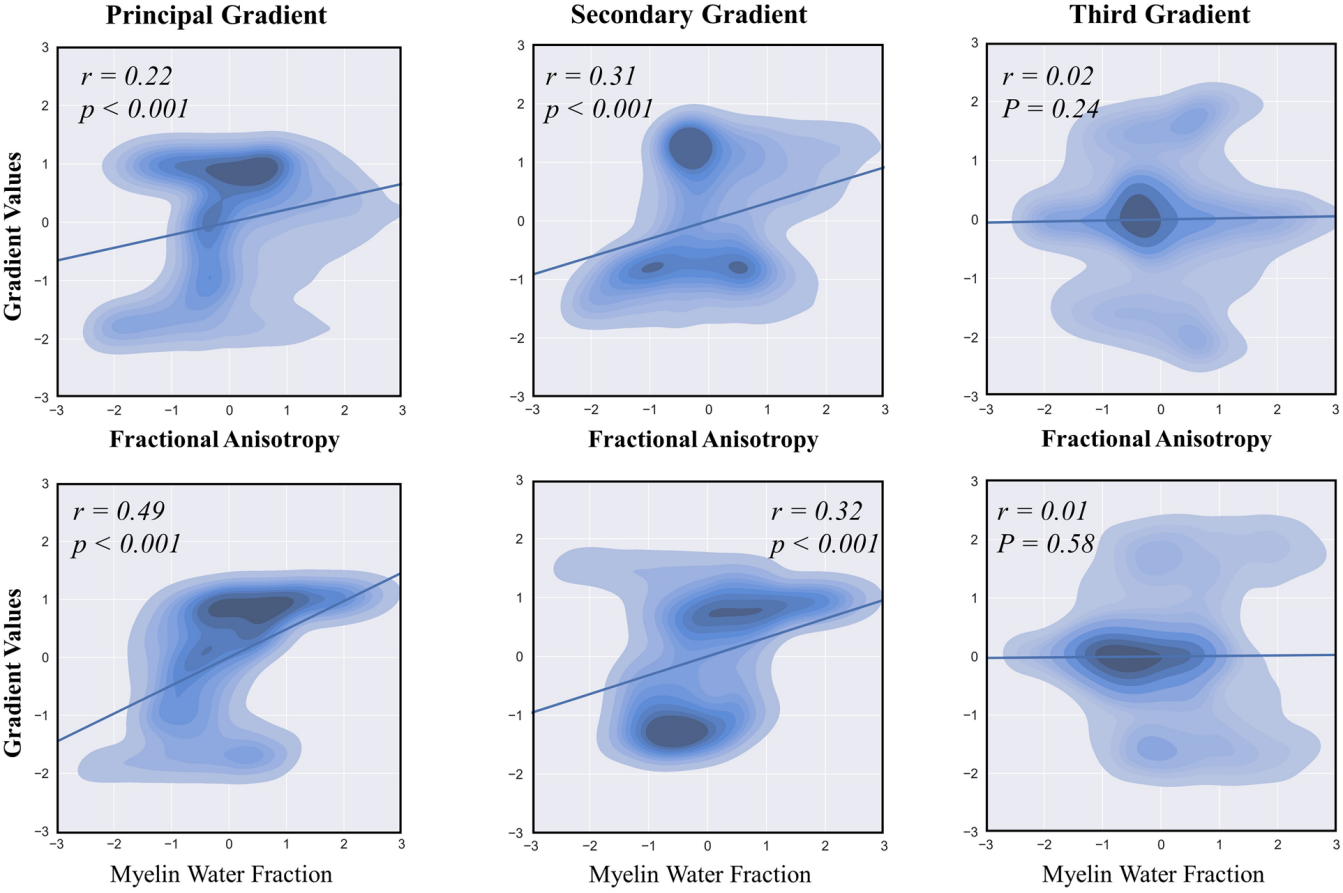
Associations between the callosal gradients and structural properties. To assess the association between callosal gradients and callosal microstructure, we extracted the fractional anisotropy (FA) and myelin water fraction (MWF) intensities at each voxel of the corpus callosum. Spatial correlation analysis indicated that FA had the highest correlation with the secondary gradient, while MWF had the highest correlation with the principal gradient.

To bridge the local microstructural properties of the corpus callosum with macroscale connectivity information in vivo, we related the functional connectome gradients to FA and MWF. This analysis revealed diverging associations with callosal FA, which strongly correlated with the principal and secondary gradients (r = 0.22 and 0.31, p < 0.001), but less strongly correlated with the third gradient (r = 0.02, p = 0.24) (Fig. 8). We also observed a stronger correlation in the secondary gradient than in the principal gradient (Steiger’s z = 4.22, p < 0.001), suggesting that the secondary gradient is most closely associated with callosal microstructural architecture. The correlations between callosal functional connectivity gradients and MWF revealed that both the principal and secondary gradients were significantly correlated with callosal MWF (r = 0.49 and 0.32, p < 0.001,Fig. 8), whereas the third gradient was not (r = 0.01, p = 0.58). The principal gradient had a stronger correlation than the secondary gradient (Steiger’s z = 7.97, p < 0.001), suggesting that the principal gradient is most closely related to the myelin density of the corpus callosum. Overall, our findings indicate that the primary and secondary gradients are significantly related to the structure of the corpus callosum, thus emphasizing the consistency between function and structure.

### 3.6 Reliability and reproducibility

**Fig. 9.**
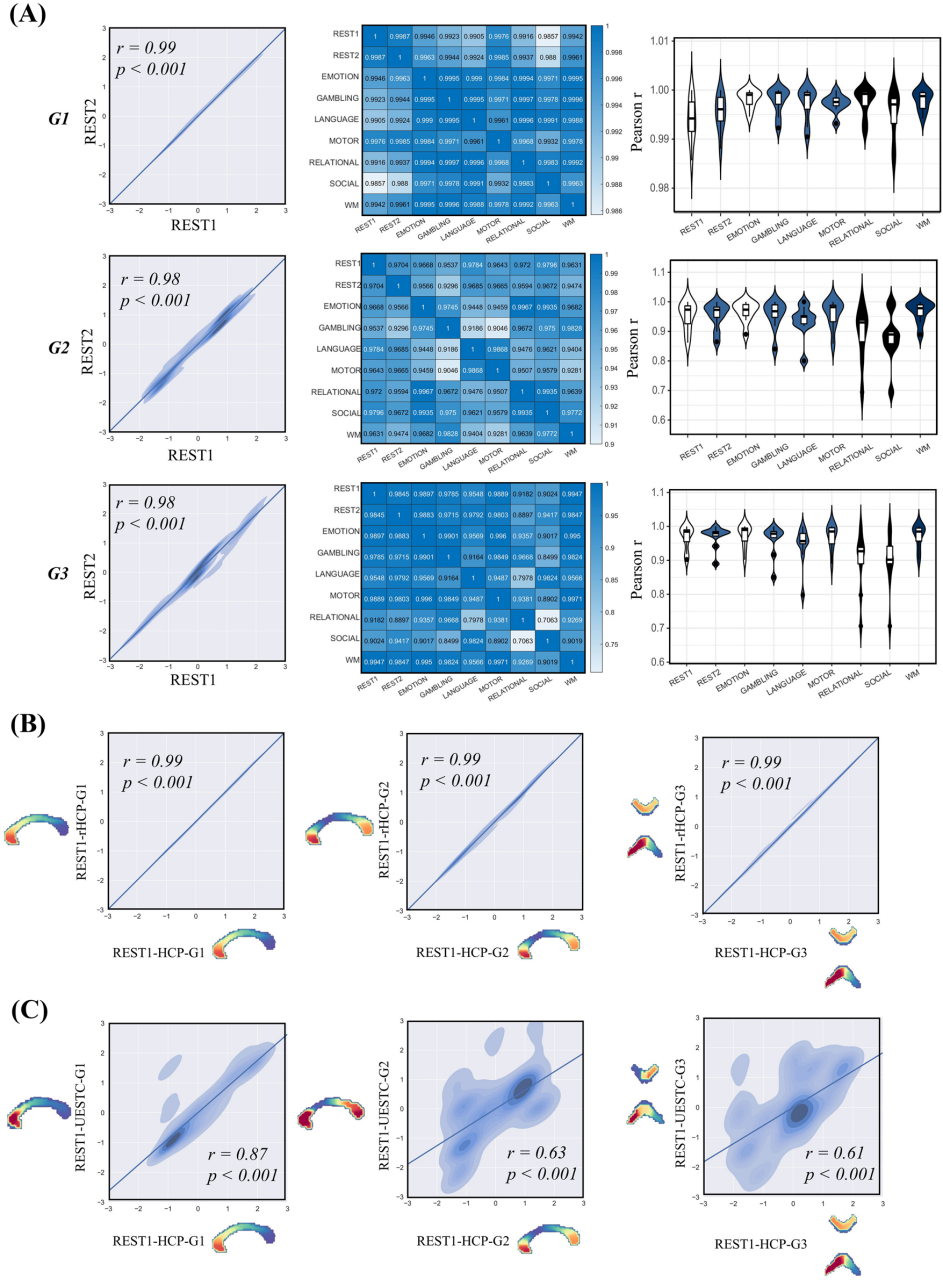
Test/retest stability of callosal functional connectivity gradients and reproducibility. (A) The group-level gradients were consistent in the HCP dataset, including the resting states (first column) and cross-task scanning conditions (second column). The average gradient similarity across tasks is greater than 0.9 (third column). The repeatability of the functional connectivity gradient of the corpus callosum was tested in the rHCP (B) and UESTC datasets (C). These results show that callosal gradients are highly stable and reproducible.

The validation analysis consisted of assessments of the group-level gradient reliability and reproducibility across scanning conditions in the main dataset, and its reproducibility in two other retest datasets. The group-level gradients were consistent across the different scans in the HCP dataset, demonstrating excellent test/retest stability (Fig. 9A). When spatially correlating gradient patterns between the HCP and retest datasets, significantly positive correlations (r = 0.99 and 0.89, respectively, in the rHCP and UESTC for the principal gradient; r = 0.99 and 0.63, respectively, for the secondary gradient; r = 0.99 and 0.61, respectively, for the third gradient; all p < 0.001) demonstrated excellent stability in all three of the gradients derived from callosal functional connectomes (Fig. 9).

## 4 Discussion

The present study was the first to investigate the functional organizational principles of the corpus callosum. We identified three gradients that gradually changed along the PA, DV, and LR axes and revealed the principal callosal gradient of functional connectivity along the cortical hierarchy. We also demonstrated that functional connectivity between the corpus callosum and cortex increased with increasing principal gradient. The secondary gradient had an N-shaped transition pattern from primary functions to higher-order cognitive functions. Additionally, these two gradient maps exhibited correlations with white matter microstructures, including FA and MWF, thus highlighting the coupling of corpus callosum function to structure. The third gradient showed an inverted V-shaped pattern, which likely reflects the separation of the left and right hemispheres and may provide new perspectives into the laterality of brain hemispheric function. The task-state fMRI findings revealed that task load enhanced the global synchronization of functional activities between the corpus callosum and the cortex, with stronger functional connectivity to the right hemisphere than the left. Moreover, an increased task load resulted in a stronger correlation between callosal functional connectivity and cognitive behavioral scores. Overall, our findings provide new and fundamental insights into the functional organization of the human corpus callosum.

Functional connectivity matrices contain multiple overlapping organizational patterns (Haak et al., 2018;Jbabdi et al., 2013), which may obscure true functional hierarchy. The gradient mapping method was therefore used in the present study, in an attempt to disentangle the overlapping connectopies that can coexist in a single region (Haak et al., 2018). The observed gradients exhibited discernible functional significance across multiple dimensions, because the connectome’s spatial configuration was able to be comprehensively depicted through a series of gradients. These gradients effectively illustrated the continuous transitions between various regions, thereby surpassing the limitations of a single dimension (Yang et al., 2020). To elucidate the organization of these gradients, we generated functions along the main axes of a callosal structure using an automatic procedure before sampling the gradient values as a function of their position along them (Drori et al., 2022). Three axes, relating to the spatial positions of the corpus callosum, were obtained. Interestingly, the gradients exhibited the exact spatial organization patterns corresponding to the three axes. The principal gradient corresponded to the PA axis of the corpus callosum, the secondary gradient corresponded to the DV axis, and the third gradient corresponded to the LR axis. These gradients suggest the existence of overlapping spatial patterns within the functional connectivity of the corpus callosum; decomposing these patterns may thus aid in a more comprehensive exploration of the functional organization of the corpus callosum.

The principal gradient may help functional diversity emerge across the cortex. It has been suggested for decades that the cortex may be organized in gradients (Nieuwenhuys, 2013). Recently, researchers have identified the existence of cortical gradient patterns through investigations of functional connectivity (Dong et al., 2022;H. C. Li et al., 2022;Margulies et al., 2016), cell types (Kim et al., 2017), receptors (Froudist-Walsh et al., 2023;Goulas et al., 2021), and gene expression (Burt et al., 2018). These studies have identified that cortical gradients vary along an axis that aligns with cortical hierarchy but have not explored the underlying mechanisms. Given the indispensable role of the corpus callosum in facilitating the transmission of cortical neural activities (Tomasch, 1954), we analyzed its functional activities in the present investigation; we identified a callosal principal gradient that increased along the cortical hierarchy. The principal gradient extended from the posterior part of the corpus callosum toward the anterior part; the splenium of the corpus callosum comprised fibers that projected to lower-order areas (e.g., occipital and inferior temporal regions), whereas the genu of the corpus callosum connected higher-order areas (i.e., prefrontal regions) (Huang et al., 2005). The functional connectivity pattern between the corpus callosum and cortex showed a similarly progressive pattern, moving from the posterior corpus callosum splenium to the anterior corpus callosum genu, corresponding to lower-order to higher-level regions. This exactly aligned with the cortical hierarchy uncovered by the cortical functional gradients (Margulies et al., 2016), implying that the corpus callosum is involved in functional activities of the cortex. Furthermore, we revealed that functional connectivity between the corpus callosum and cortex increased with increasing principal gradient. This increased integration between the corpus callosum and cortex may enable neurons in higher cortical regions to function more flexibly. Spatially, we demonstrated that the k-means clustering algorithm was able to divide the corpus callosum into functional subregions based on the principal gradient; these subregions highly overlapped with anatomy-based subregions, indicating that the gradient map of the corpus callosum has a spatial layout that is consistent with its previously hypothesized anatomical–functional organization (Reyes et al., 2020). In addition, the principal gradient was observed to correspond to the intrinsic callosal geometry, suggesting that differences in distance can alter discordance among regional connectivity patterns. We also identified a positive correlation between myelination and principal gradient values in the corpus callosum, suggesting that the anterior corpus callosum, with its higher gradient values, may have more myelination. Myelin electrically insulates axons to allow the fast propagation of nerve impulses (Lim & Rasband, 2020). Consequently, myelination plays a pivotal role in maintaining neuronal function, and disruptions in this process can give rise to severe and debilitating diseases (Jiang et al., 2023;Nave & Werner, 2014). Together, our findings indicate that the corpus callosum may be fundamental for establishing and maintaining the functional diversity and hierarchy that are observed in cortical regions.

In the present study, the second callosal gradient provides further evidence for the coupling of function and structure of the corpus callosum. The secondary gradient separated the ventral attention network and the DMN. The corpus callosum regions connected with the DMN were distant from the regions connected with the ventral attention network; this finding is consistent with the idea that the DMN and ventral attention network are two opposing brain systems (Fox et al., 2005). Activity in these two networks is often anticorrelated, in line with their opposing roles in cognition (Kelly et al., 2008). Notably, the DMN is active when attention is not focused on the external world (Andrews-Hanna et al., 2014). In the current study, however, we revealed a significant correlation between FA and secondary gradient values in the corpus callosum. Consistent with the organization of the secondary gradient, the FA values increased from early visual regions to later visual regions, decreased toward the middle segments, and then rose again toward the frontal pole segment (Friedrich, Fraenz, et al., 2020). Additionally, the FA in the corpus callosum exhibited a strong correlation with axon density (Friedrich, Fraenz, et al., 2020), which indicates that the organizational pattern of the secondary gradient is similar to that of axon density in the corpus callosum. The second callosal gradient thus accurately reflects function–structure coupling.

The third gradient may contribute to the functional lateralization of the cerebral hemisphere. The third gradient starts from the left of the corpus callosum and spreads to the right. It can therefore easily be related to the basic function of the corpus callosum: interhemispheric information transfer (Roland et al., 2017). Previous research has revealed that mostly homotopic regions of the hemispheres are connected by callosal fibers (Putnam et al., 2008), and that interhemispheric functional connectivity is lost shortly after the complete transection of the corpus callosum (Roland et al., 2017). These findings are in line with our results, that the ROIs in the third gradient have similar cortical functional connectivity patterns to its symmetric ROIs. However, a progressive—rather than symmetrical—organization pattern of this gradient was identified in the corpus callosum, which may help to elucidate the potential role of the corpus callosum in functional hemispheric asymmetries. There is a long history of the corpus callosum as an underlying factor of functional hemispheric asymmetries (Ocklenburg et al., 2016). In the present study, functional connectivity between the corpus callosum and the right cortex was greater than that of the left, and this discrepancy in connectivity was exacerbated under task conditions. This provides clues to the balancing properties of the corpus callosum in processing recourses between hemispheres. From an evolutionary perspective, the corpus callosum is thought to play an integral role in the development of higher-order cognitive functions and hemispheric specialization (Doron & Gazzaniga, 2008). Studies in split-brain patients indicate that an absence or loss of corpus callosum integrity contributes to impairments in sensory and cognitive integration (Roland et al., 2017). Recent studies have reported decreased lateralization with age; tasks that are strongly lateralized in young adults can become bilateral in older brains (Addamo et al., 2007). Our investigation of the callosal gradient therefore deepens our understanding of the involvement of the corpus callosum in interhemispheric information transmission, and indicates that further exploration is needed.

In particular, functional connectivity between the corpus callosum and cortex was significantly stronger in the task state than in the resting state. Task performance commonly results in increased connectivity among the cortical networks recruited by the task. For example, Shine et al. illustrated a more tightly integrated relationship among the fronto-parietal, dorsal attention, cingulo-opercular, and visual networks when participants engaged in a 2-back working memory task (Shine et al., 2016). Moreover, previous studies have suggested that there is increased connectivity between the dorsal attention and visual networks during attention to visual stimuli (Kwon et al., 2017;Spadone et al., 2015). Similarly, we observed increased callosal functional connectivity across different task load conditions in the current study. Given that cortical nerve signals are transmitted through white matter fiber bundles, one possible explanation for this phenomenon is that, when two brain regions interact (especially across hemispheres), the corpus callosum acts as an important hub and has increased functional connectivity with the two brain regions. Other investigations have revealed that domain-specific across-network connectivity increases in association with task performance (Gonzalez-Castillo & Bandettini, 2018). Correspondingly, we also demonstrated that task loads led to significantly increased correlations between corpus callosum functional connectivity and cognitive behavioral scores, indicating that task-related changes in callosal functional connectivity strongly contribute to brain activation during cognitive task performance. The results of the present study provide evidence for a role of the corpus callosum in cognitive behavior.

The current study had several limitations. First, we did not obtain structural connectivity results for the corpus callosum; however, other studies have demonstrated the DTI-based connectivity of the corpus callosum (Huang et al., 2005). Second, we only analyzed the group-level functional connectivity gradients of the corpus callosum, and did not evaluate individual-level functional connectivity gradients. However, our individual-level callosal gradients showed poor intra- and inter-subject consistency, unlike the results of previous studies in gray matter brain regions (Dong et al., 2022;Yang et al., 2020). This finding may be because BOLD signals in white matter have a relatively low signal-to-noise ratio and are more susceptible to other factors. In addition, uncertainty remains regarding whether observed white-matter signals truly reflect neuron-related activity, because white matter has very few postsynaptic potentials that can give rise to BOLD signals (Logothetis et al., 2001). The same white matter locations may also combine signals from different functional systems because of the crossing of white matter tracts. Fortunately, we obtained stable callosal gradient results at the group level and validated these findings across different scanning conditions and in independent datasets, thus providing strong evidence for the functional signature of white matter BOLD signals in the present study. In addition, significant task-specific HRFs were detected in white matter compared to gray matter, so the standard HRF needs to be modified to accurately characterize activation in white matter when analyzing white matter BOLD in the task state (M. Li et al., 2019). Finally, considering the association between the corpus callosum and cognitive behavior, future explorations of correlations between the corpus callosum and psychiatric diseases, as well as investigations of its clinical significance, are potential directions for further research.

In conclusion, our study provides the first comprehensive functional gradient map of the corpus callosum, thus elucidating its underlying functional and structural architecture. We revealed three inherent functional patterns of the corpus callosum using gradient mapping. By integrating the three gradients with anatomical, resting-state, and task-state functional connectivity data, we revealed the potential role of the corpus callosum in the cortical hierarchy, structural and functional laterality. Task load was related to stronger functional connectivity between the corpus callosum and cortex, along with an increased correlation with cognitive behavioral scores. By unraveling the functional intricacies of the human corpus callosum, our study contributes new insights into the potential functions of this crucial brain structure, especially in the context of neurological disorders.

## Supplementary Material

Supplementary Material

## Data Availability

The Human Connectome Project dataset was publicly available athttps://db.humanconnectome.org/study/hcp-young-adult. The atlas of the average myelin water fraction distribution in the adult human brain can be available athttps://sourceforge.net/projects/myelin-water-atlas/. The codes for gradient mapping are openly available athttps://github.com/yetianmed/subcortex. The following additional software packages used for this study are freely and openly available: Data Processing Assistant for Resting-State fMRI:http://rfmri.org/DPARSFand SPM12:http://www.fil.ion.ucl.ac.uk/spm/software/spm12. Other information is available upon reasonable request.
