# Supplementary material for: Functional organization of the human corpus callosum unveiled with BOLD-fMRI gradients
Source: Imaging Neurosci (Camb). 2024 Mar 25;2:imag-2-00115. doi: 10.1162/imag_a_00115 (PMC12247586; doi:10.1162/imag_a_00115)
Supplement: Supplementary Material [file imag_a_00115-supp.pdf]

Supplementary Material For

Functional organization of the human corpus callosum unveiled with BOLD-fMRI gradients

Huan Huang[1,2†], Yuchao Jiang[3,4†], Hechun Li[1,2], Hanxi Wu[1,2], Xiaorong Feng[1,2], Jinnan Gong[1,2], Sisi Jiang[1,2], Dezhong Yao[1,2], Cheng Luo[1,2*]

[1]The Clinical Hospital of Chengdu Brain Science Institute, MOE Key Lab for Neuroinformation, School of Life Science and Technology, University of Electronic Science and Technology of China, Chengdu 611731, P. R. China

[2]Research Unit of NeuroInformation, Chinese Academy of Medical Sciences, 2019RU035, Chengdu, P. R. China

[3]Institute of Science and Technology for Brain-Inspired Intelligence, Fudan University, Shanghai, China

[4]Key Laboratory of Computational Neuroscience and Brain-Inspired Intelligence (Fudan University), Ministry of Education, China

[†]These authors contributed equally to this work.

[*]Address correspondence to: Cheng Luo; chengluo@uestc.edu.cn

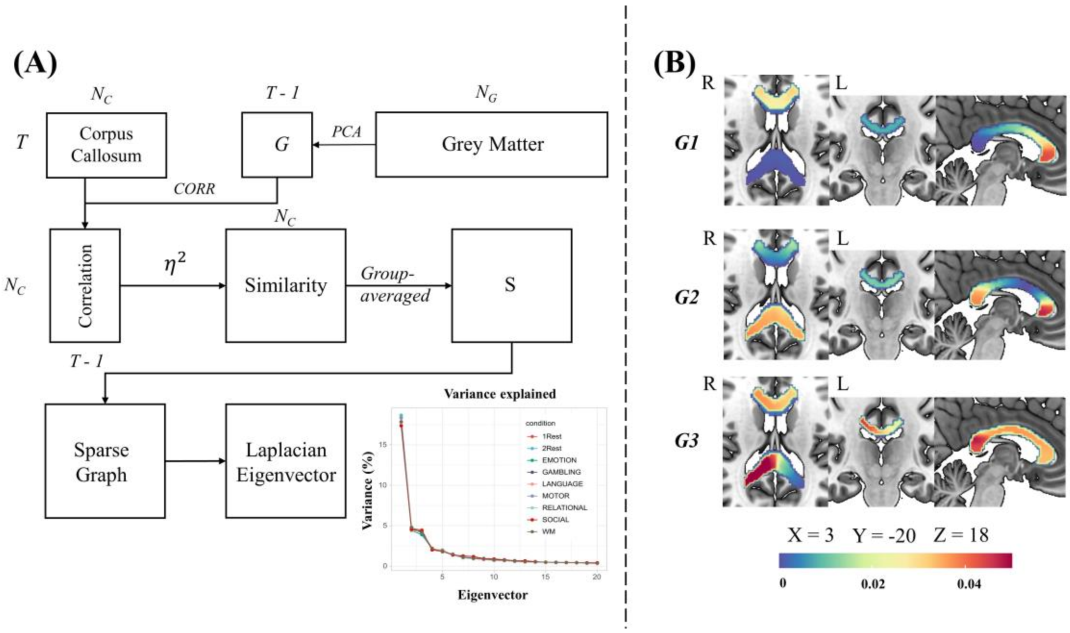

Fig. S1 A schematic of functional connectivity gradient mapping within corpus callosum. (A) First, PCA was used to reduce the dimensionality of the time series matrix of the gray voxel, and then the Pearson correlation coefficients between the corpus callosum and the dimensionality reduction matrix (G) were calculated. The similarity in connectional fingerprints between pairs of callosal voxels was measured using the η^2 coefficient. Pairs of voxels with similar fingerprints connected to similar brain regions. Subsequently, the similarity matrices of group-level were analyzed. The group-level similarity matrix (S) represents a group consensus for all participants. The similarity matrix was transformed into a sparse graph with an adjacency matrix. The graph nodes correspond to callosal voxels. Laplacian eigenvalues and eigenvectors were computed for the graph Laplacian. (B) The callosal functional gradient maps at the group level correspond to REST1 scanning conditions. G1 describes an anterior/posterior gradient, G2 describes a ventral/dorsal gradient, and G3 describes a right/left gradient. N_C, number of callosal voxels; N_G, number of gray matter voxels; T, number of time frames; G1, principal gradient; G2, secondary gradient; G3, third gradient.

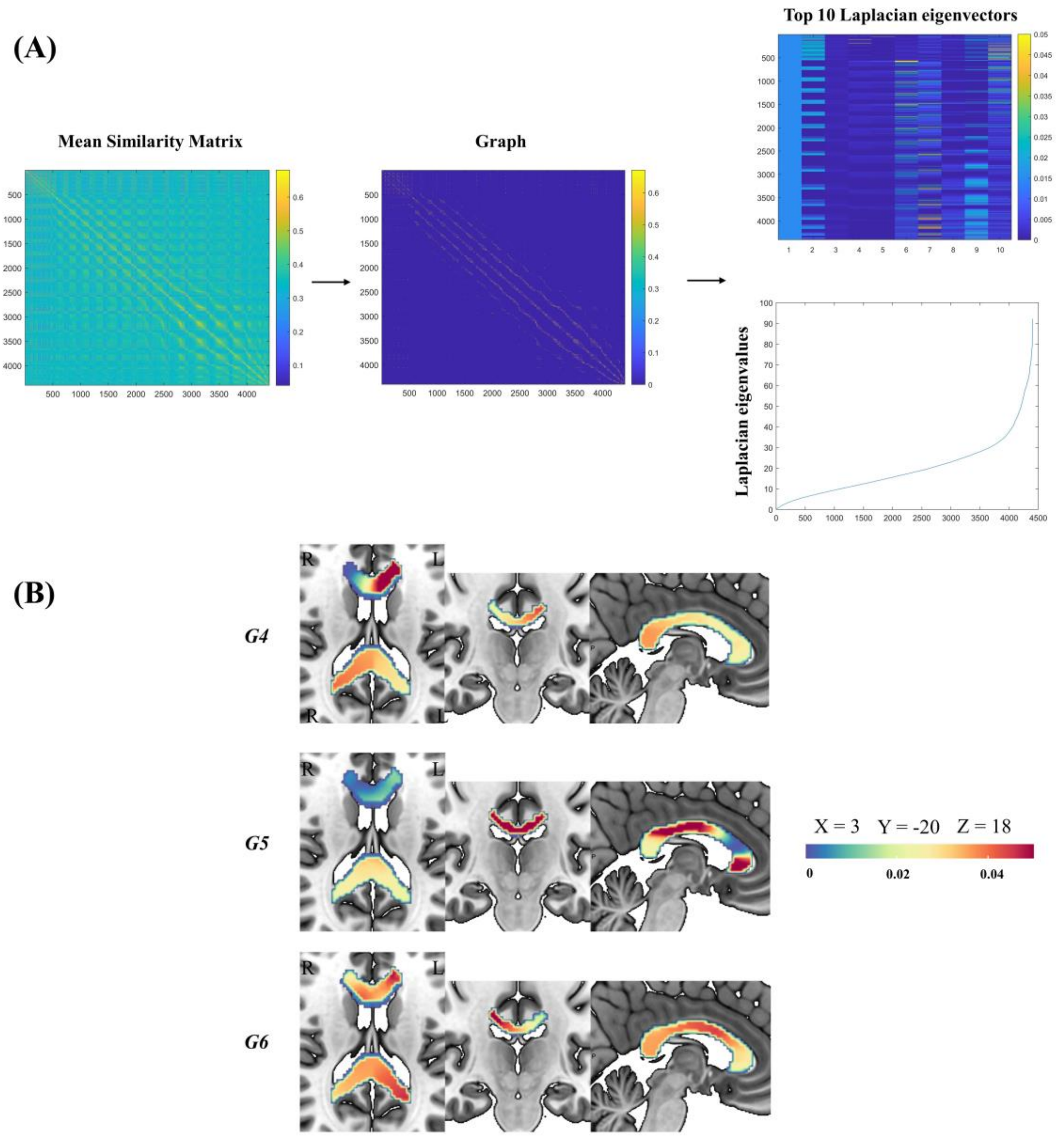

Fig. S2 schematic of Laplacian matrix decomposition. (A) The mean similarity matrix (left) was transformed into a sparse graph with an adjacency matrix (middle). Then, the Laplacian eigenvalues and eigenvectors were computed for the graph Laplacian (right). The graph above shows the eigenvectors corresponding to the first 10 smallest eigenvalues, and the graph below shows the distribution of all eigenvalues. (B) The callosal functional gradient 4, 5 and 6 maps at the group level under REST1 scanning conditions. G4, fourth gradient; G5, fifth gradient; G6, sixth gradient.

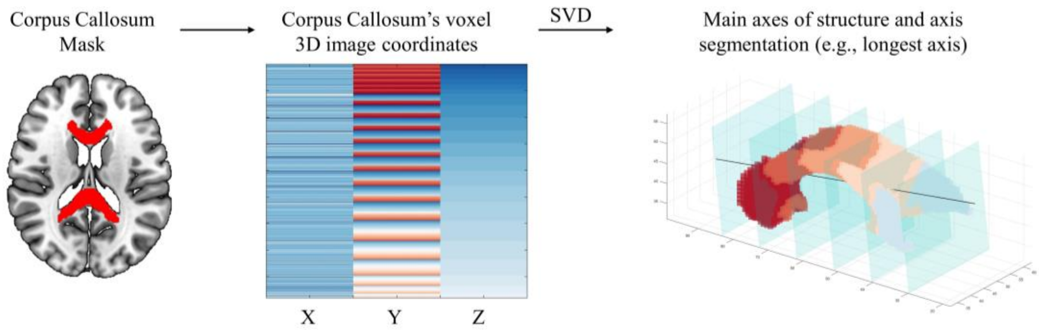

Fig. S3 The flow diagram of ROI-based axes for the corpus callosum. To explore how the functional gradient varies along the spatial position of the corpus callosum, we used the automatic axis computation algorithm to generate ROI-based axes for the corpus callosum. The process includes the following two steps. First, the algorithm computes the SVD of the ROI's voxel 3D image coordinates to find the main three orthogonal axes (i.e., the eigenvectors) of the structure (middle). Second, for each of the three main axes, we segment the ROI with equal spacing. For this purpose, we define the data edges as the two hyperplanes defined by the two extreme data points of the data for the axis and by the axis as normal to the plane. The data are then segmented by $n - 1$ parallel hyperplanes equally spaced between the two data edges. Voxels are then classified into n segments based on criteria of distance from planes (right). In our study, we chose $n = 5$.

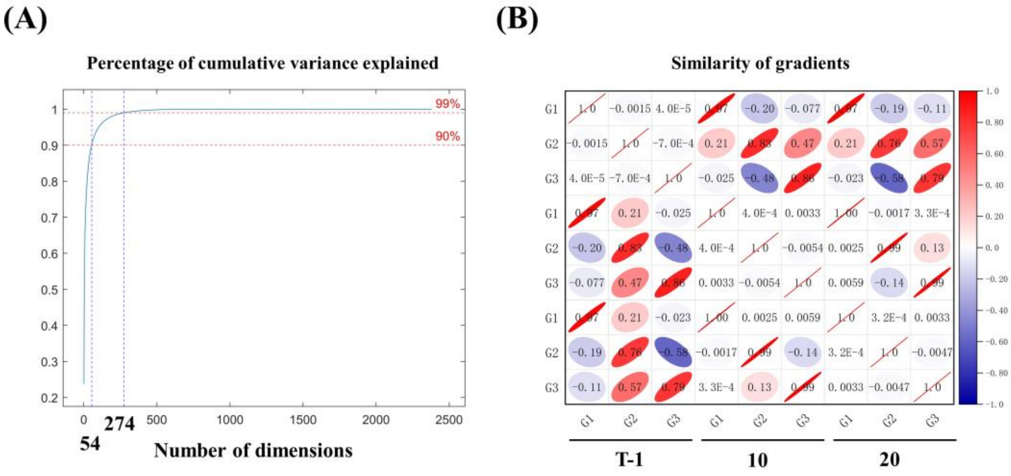

Fig. S4 To evaluate the effect of the number of dimensionality reduction in grey matter time series on the gradient. (A) Percentage of cumulative variance explained for the grey matter time series. The results show that the cumulative percentage of 54 and 274 dimensions achieved 90% and 99% respectively, leading us to choose that the number of dimensions should be greater than or equal to 54. (B) To further evaluate the effect of a smaller number of dimensions on the gradient. We calculated the corpus callosum gradients with dimensionality reduction of 10 and 20, respectively, and then calculated the correlation with the gradients with dimensionality reduction of T-1. The results showed that the first three gradients of the corpus callosum had good reproducibility at smaller dimensions (r=0.7~0.8), but were less testability than those at T-1 dimensions (r>0.95).

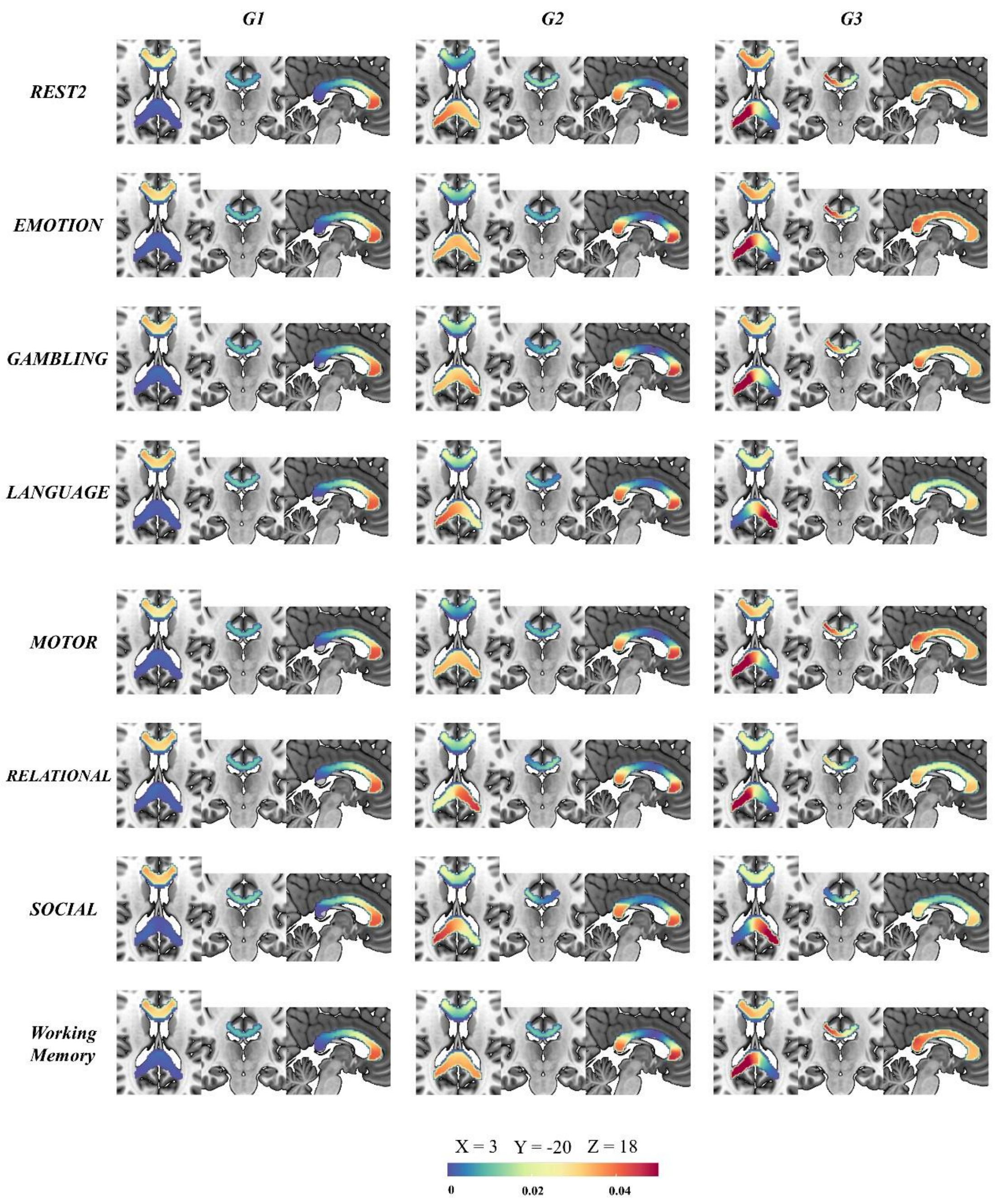

Fig. S5 The callosal functional gradient maps corresponding to all scanning conditions. G1 describes an anterior/posterior gradient, G2 describes a ventral/dorsal gradient, and G3 describes a right/left gradient. G1, principal gradient; G2, secondary gradient; G3, third gradient.

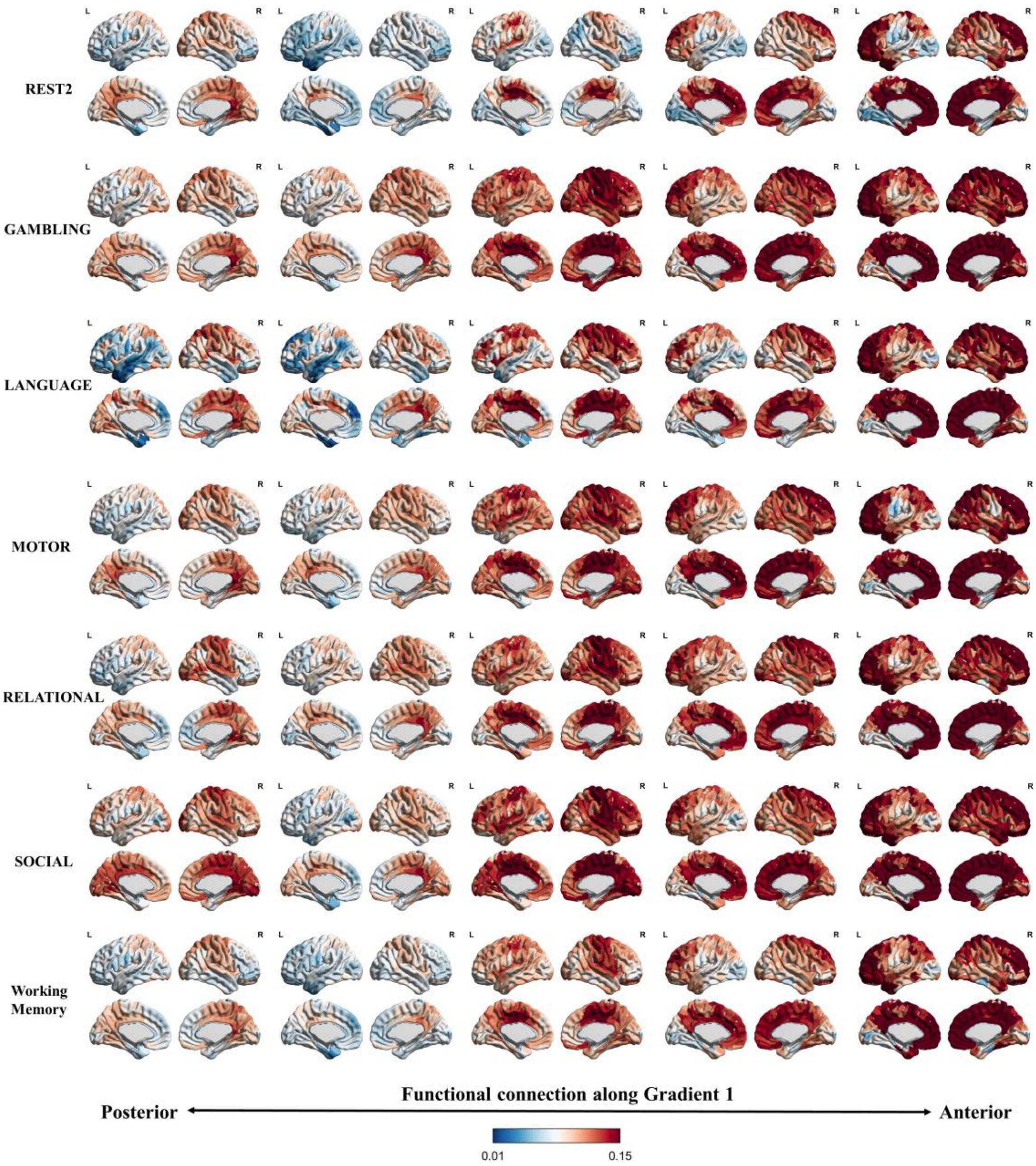

Fig. S6 Callosal connectivity patterns along the callosal principal gradient in the different scanning conditions. The black arrows represent the approximate location of the ROIs along the callosal posterior/anterior (PA) axis. Gradient 1, principal gradient.

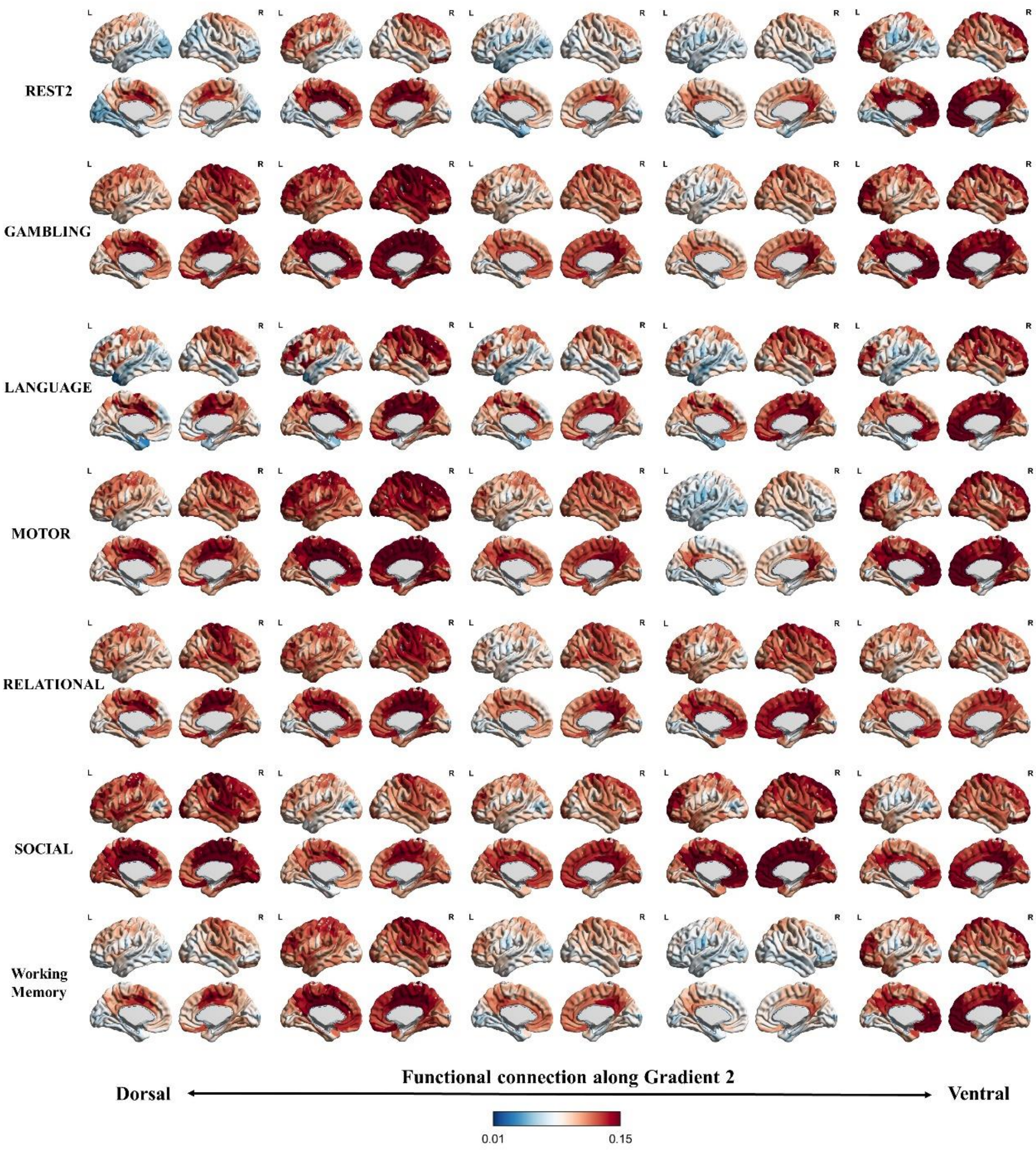

Fig. S7 Callosal connectivity patterns along the callosal secondary gradient in the different scanning conditions. The black arrows represent the approximate location of the ROIs along the callosal dorsal/ventral (DV) axis. Gradient 2, secondary gradient.

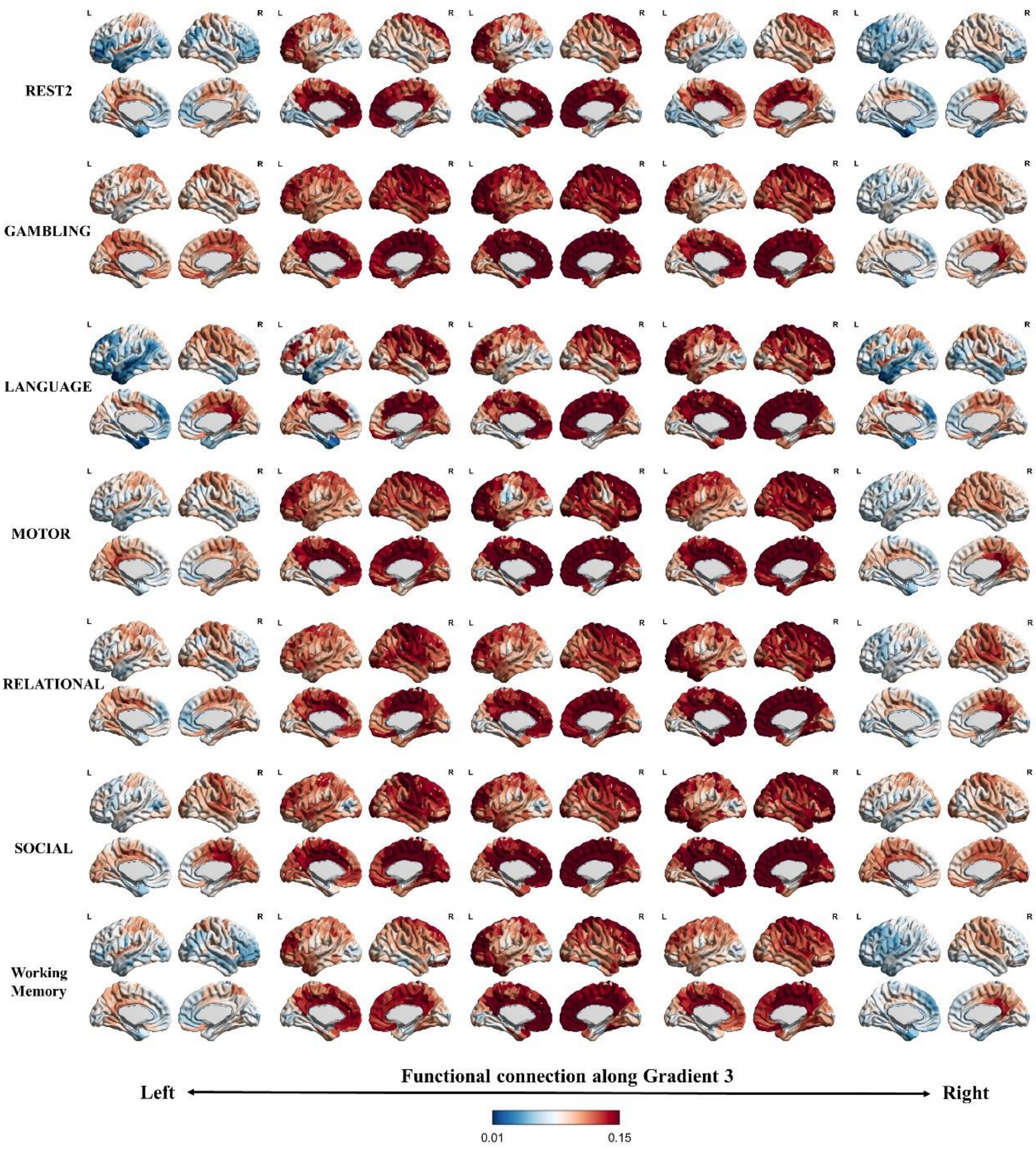

Fig. S8 Callosal connectivity patterns along the callosal third gradient in the different scanning conditions. The black arrows represent the approximate location of the ROIs along the callosal left/right (LR) axis. Gradient 3, third gradient.

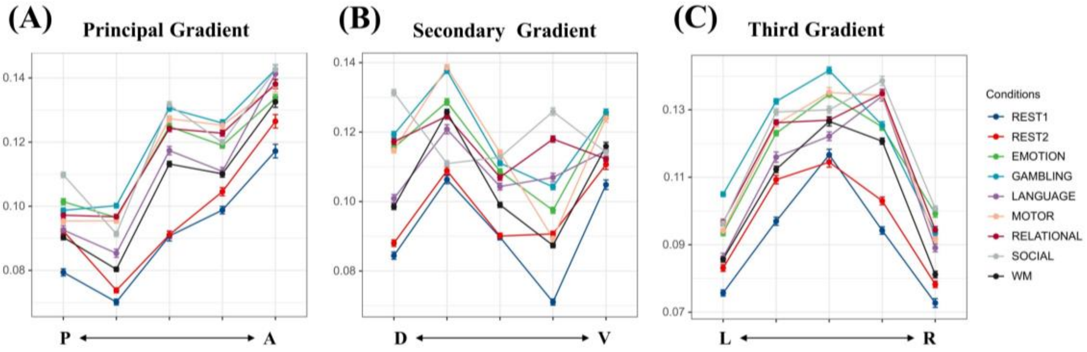

Fig. S9 The average functional connections between the ROIs along callosal functional gradients and the cortical in the different scanning conditions. (A) Along the principal gradient, functional connections between the corpus callosum and cortex generally showed a gradual increase from posterior to anterior. (B) Along the secondary gradient, the functional connections between the corpus callosum and cortex generally increase from the ventral to the dorsal, then decrease, and then increase again, showing an 'N' shape. (C) Along the third gradient, the functional connections between the corpus callosum and cortex generally increase first and then decrease from the left to right, and reach a peak in the central sagittal plane of the corpus callosum, showing an inverted triangle shape with left and right symmetry. Overall, the functional connection between the corpus callosum and cortex was similar in different scanning conditions.

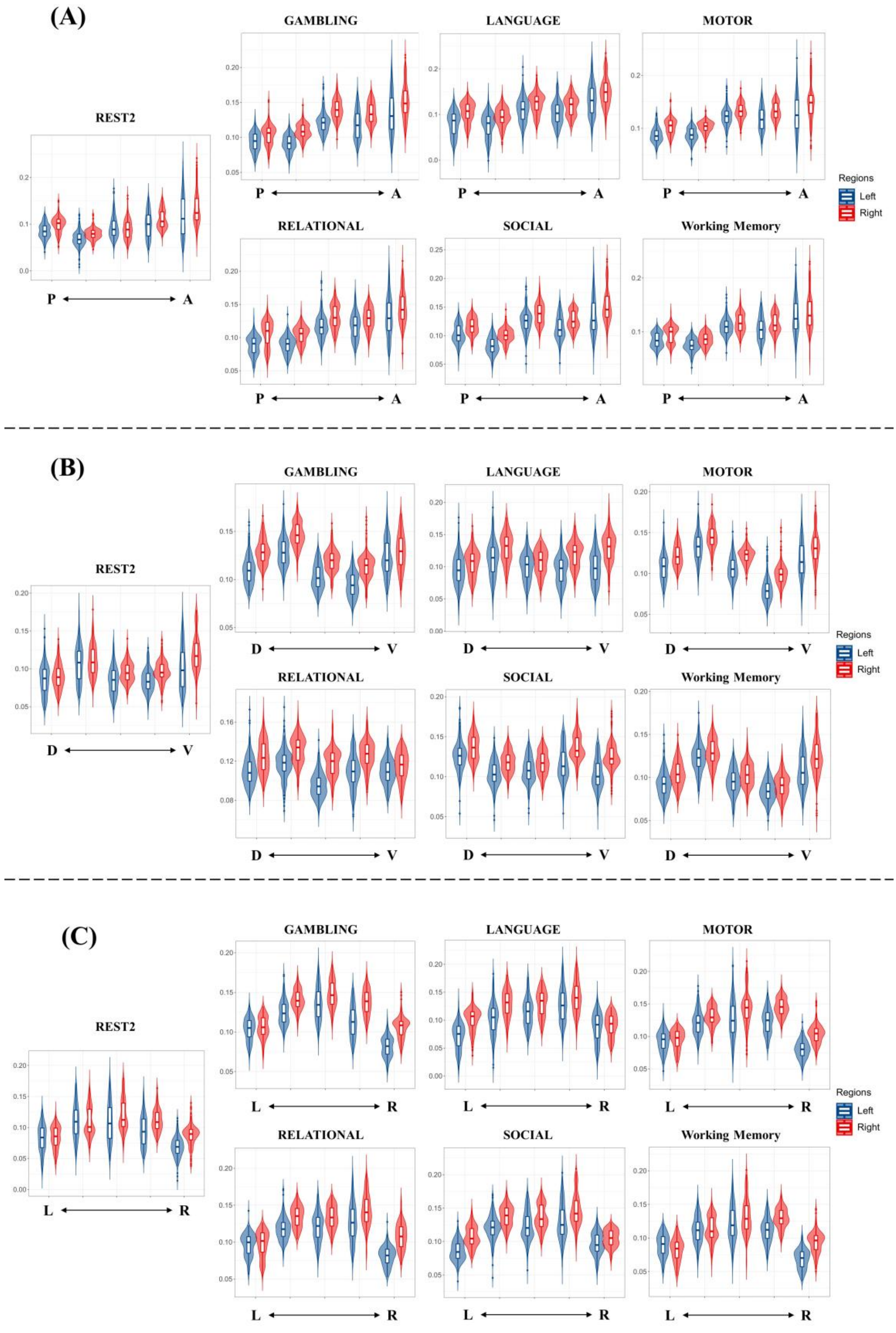

Fig. S10 The average functional connectivity between the ROIs along callosal functional gradients and the left and right cortical in the different scanning conditions. (A) principal gradient, (B) secondary gradient, (C) third gradient.

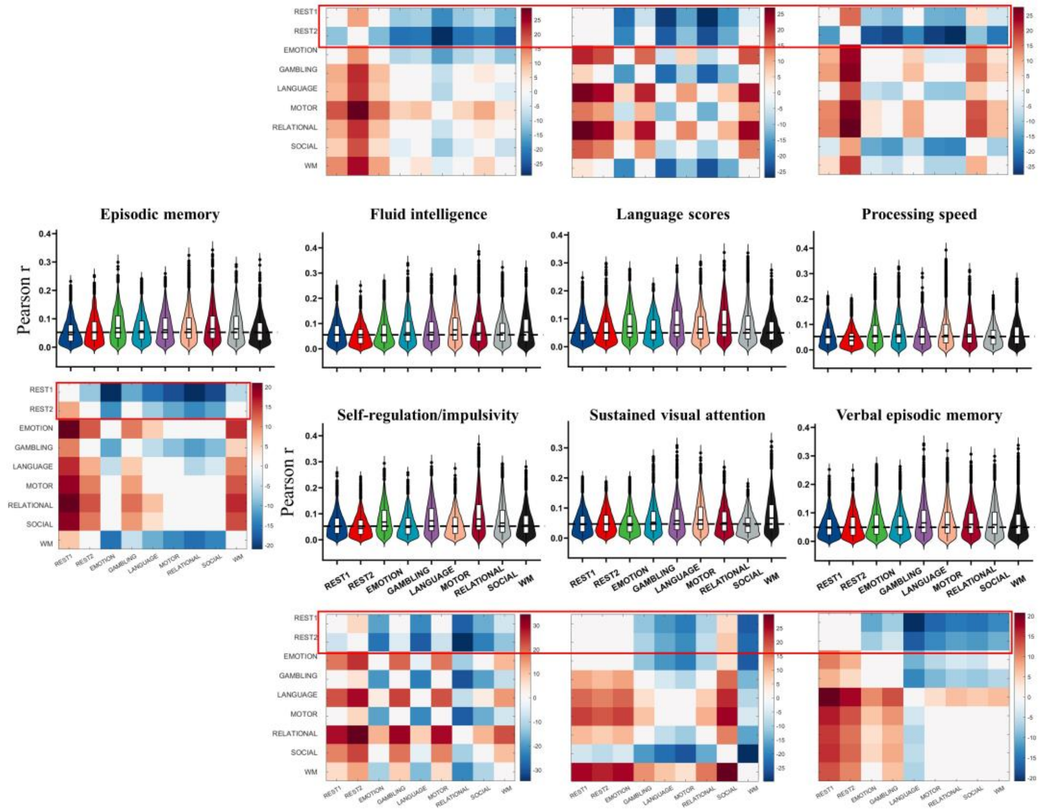

Fig. S11 Relating callosal-cortical functional connectivity to behavioral scores in all scanning conditions. The violin plot shows the correlation between callosal functional connectivity and behavioral scores. Absolute values of Pearson's correlation coefficients are shown for each callosal voxel. The black dotted line indicates the maximum value of the averaged correlation in REST1 and REST2. The corresponding heat map shows the T-value map obtained by paired sample T-test for the correlation coefficients across all scanning conditions. As indicated by the red box, the functional connectivity between the corpus callosum and the cortex was more strongly correlated with the behavior in the task scanning conditions.

(A)

Voxel Number (p<0.05)	Episodic memory	Executive functions	Fluid intelligence	Language	Processing speed	Self-regulation/impulsivity	Spatial orientation	Sustained visual attention	Verbal episodic memory	Working memory
REST1	212	140	337	280	202	257	72	106	164	237
REST2	357	119	133	309	82	158	137	111	257	166
EMOTION	238	75	202	329	209	293	204	22	203	473
GAMBLING	133	109	423	104	260	72	239	155	183	145
LANGUAGE	114	120	194	335	74	297	129	108	278	113
MOTOR	301	231	509	303	369	80	429	252	354	202
RELATIONAL	200	138	201	322	137	449	318	21	89	218
SOCIAL	318	149	251	452	38	476	264	3	282	230
WM	116	192	493	160	176	224	271	370	337	102

(B)

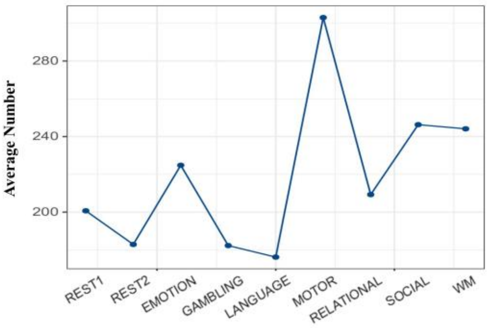

Fig. S12 The number of callosal voxels that were significantly associated with behavioral scores in all scanning conditions. (A) Tables show the count of voxels with $p < 0.05$ for all scanning conditions and behavioral scores. (B) The average number of corpus callosum voxels is significantly associated with cognition under different task conditions ($p<0.05$, uncorrected). In general, the number of voxels in the corpus callosum in the task state was higher than that in the resting state.

Table S1. The maximum correlation of the eigenvectors between the REST1 and other scanning conditions

Rest1 (Template)	**Eigenvector1**	**Eigenvector2**	**Eigenvector3**
Rest2	1(0.99)	2(0.99)	3(0.98)
EMOTION	1(0.99)	2(0.97)	3(0.99)
GAMBLING	1(0.99)	3(0.96)	2(0.98)
LANGUAGE	1(0.99)	2(0.93)	3(-0.95)
MOTOR	1(0.99)	2(0.99)	3(0.99)
RELATIONAL	1(0.99)	3(0.90)	2(0.92)
SOCIAL	1(0.99)	3(0.86)	2(-0.90)
WORKING MEMORY	1(0.99)	3(0.98)	2(0.99)

Each row of the table represents the order of the eigenvector with the highest correlation coefficient to the different eigenvectors in the template in the specified scanning condition.

Table S2. PCA inputs for behavioral scores

Domain	Measure	Short name
**Episodic memory**		
	Picture Sequence Memory	PicSeq_Unadj
**Executive functions**		
	Dimensional Change Card Sort Test	CardSort_Unadj
	Flanker Inhibitory Control and Attention Test	Flanker_Unadj
**Fluid intelligence**		
	Penn Progressive Matrices: Number of Correct Responses	PMAT24_A_CR
	Penn Progressive Matrices: Total Skipped Items	PMAT24_A_SI
	Penn Progressive Matrices: Median Reaction Time for Correct Responses	PMAT24_A_RTCR
**Language**		
	Oral Reading Recognition Test	ReadEng_Unadj
	Picture Vocabulary Test	PicVocab_Unadj
**Processing speed**		
	Pattern Comparison Processing Speed Test	ProcSpeed_Unadj
**Self-regulation/impulsivity**		
	Delay Discounting	DDisc_AUC_200
**Spatial orientation**		
	Variable Short Penn Line Orientation	VSPLOT_TC
	Variable Short Penn Line Orientation: Median Reaction Time Divided by Expected Number of Clicks for Correct	VSPLOT_CRTE
	Variable Short Penn Line Orientation: Total Positions Off for All Trials	VSPLOT_OFF
**Sustained visual attention**		
	Short Penn Continuous Performance Test: Median Response Time for True Positive Responses	SCPT_TPRT
	Short Penn Continuous	SCPT_SEN

	Performance Test: Sensitivity	
	Short Penn Continuous Performance Test: Specificity	SCPT_SPEC
	Short Penn Continuous Performance Test: Longest Run of Non-Responses	SCPT_LRNR
**Verbal episodic memory**		
	Penn Word Memory Test: Total Number of Correct Responses	IWRD_TOT
	Penn Word Memory Test: Median Reaction Time for Correct Responses	IWRD_RTC
**Working memory**		
	List Sorting Working Memory Test	ListSort_Unadj
